# Burst-Time-Dependent Plasticity Robustly Guides ON/OFF Segregation in the Lateral Geniculate Nucleus

**DOI:** 10.1371/journal.pcbi.1000618

**Published:** 2009-12-24

**Authors:** Julijana Gjorgjieva, Taro Toyoizumi, Stephen J. Eglen

**Affiliations:** 1 Department of Applied Mathematics and Theoretical Physics, University of Cambridge, Cambridge, United Kingdom; 2 Department of Neuroscience, Columbia University, New York, New York, United States of America; Université Paris Descartes, Centre National de la Recherche Scientifique, France

## Abstract

Spontaneous retinal activity (known as “waves”) remodels synaptic connectivity to the lateral geniculate nucleus (LGN) during development. Analysis of retinal waves recorded with multielectrode arrays in mouse suggested that a cue for the segregation of functionally distinct (ON and OFF) retinal ganglion cells (RGCs) in the LGN may be a desynchronization in their firing, where ON cells precede OFF cells by one second. Using the recorded retinal waves as input, with two different modeling approaches we explore timing-based plasticity rules for the evolution of synaptic weights to identify key features underlying ON/OFF segregation. First, we analytically derive a linear model for the evolution of ON and OFF weights, to understand how synaptic plasticity rules extract input firing properties to guide segregation. Second, we simulate postsynaptic activity with a nonlinear integrate-and-fire model to compare findings with the linear model. We find that spike-time-dependent plasticity, which modifies synaptic weights based on millisecond-long timing and order of pre- and postsynaptic spikes, fails to segregate ON and OFF retinal inputs in the absence of normalization. Implementing homeostatic mechanisms results in segregation, but only with carefully-tuned parameters. Furthermore, extending spike integration timescales to match the second-long input correlation timescales always leads to ON segregation because ON cells fire before OFF cells. We show that burst-time-dependent plasticity can robustly guide ON/OFF segregation in the LGN without normalization, by integrating pre- and postsynaptic bursts irrespective of their firing order and over second-long timescales. We predict that an LGN neuron will become ON- or OFF-responsive based on a local competition of the firing patterns of neighboring RGCs connecting to it. Finally, we demonstrate consistency with ON/OFF segregation in ferret, despite differences in the firing properties of retinal waves. Our model suggests that diverse input statistics of retinal waves can be robustly interpreted by a burst-based rule, which underlies retinogeniculate plasticity across different species.

## Author Summary

Many central targets in the brain are involved in the processing of information from the outside world. Before information about the visual scene reaches the visual cortex, it is preprocessed in the retina and the lateral geniculate nucleus. Connections which relay this information between the different brain targets are not determined at birth, but undergo a developmental period during which they are guided by molecular cues to the correct locations, and refined by activity to the appropriate numbers and strengths. Before the onset of vision, spontaneous activity generated within the retina plays an important role in the remodeling of these connections. In a computational and theoretical model, we used recorded spontaneous retinal activity patterns with several plasticity rules at the retinogeniculate synapse to identify the key properties underlying the selective refinement of connections. Our model shows robust behavior when applied to both mouse and ferret data, demonstrating that a common plasticity rule across species may underlie synaptic refinements in the visual system driven by spontaneous retinal activity.

## Introduction

During the development of the visual system, connections between neurons form and refine in a self-organized manner governed by various mechanisms. Initially, target neurons are contacted by multiple RGCs following gradients of molecular cues [1]–[3]. As circuits mature, visually-evoked activity maintains these connections; however, in early development when photoreceptors are functionally inactive, activity is spontaneously generated within the retina. This spontaneous activity spreads across the retina in the form of waves, and is believed to encode different cues for synapse maturation in the visual system [4]: as inappropriate connections are eliminated, appropriate connections are strengthened following Hebbian-like coincidence detection mechanisms [5],[6]. There is a long-standing, and still active, debate about the relative importance of activity-dependent mechanisms in development [7],[8]. Theoretical models can help inform this debate by evaluating hypotheses about the role of neural activity in the remodeling of connections.

One possible mechanism of coincidence detection of pre- and postsynaptic activity is that of spike-time-dependent plasticity (STDP): synaptic change is induced from pairing multiple pre- and postsynaptic spikes, firing within tens of milliseconds of each other [9],[10]. Various extensions which include triplets, quadruplets and other nonlinearities in spike integration have also been studied [11]–[13], but they commonly predict synaptic potentiation if presynaptic activity shortly precedes postsynaptic activity, and synaptic depression otherwise. One may argue that in developing systems, immature synapses are incapable of encoding information using precisely-timed spikes, but use bursts over coarser timescales [14],[15]. Butts et al. [16] recently proposed burst-time-dependent plasticity (BTDP), based on recordings at the developing retinogeniculate synapse, as an alternative to STDP. In BTDP, synaptic change is induced according to the timing of bursts over longer timescales of a second, and irrespective of the firing order of pre- and postsynaptic bursts.

To compare spike- and burst-based mechanisms in the remodeling of synaptic connections in a realistic developmental scenario, we examine the segregation of ON and OFF RGCs (which respond to light increments and decrements, respectively) onto postsynaptic neurons in the LGN. Early in development, individual LGN neurons receive inputs from 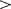
20 mixed ON and OFF RGCs; these inputs segregate such that eventually LGN neurons receive inputs from 1–3 RGCs of the same type (ON or OFF) [6],. Blocking spontaneous activity inhibits this segregation [18],[19]. Recent experiments in mouse identified a difference in the firing patterns of RGCs that might instruct ON/OFF segregation: at P12, cells of the same type fire together, but OFF RGCs fire about a second after ON RGCs [20]. This asynchrony differs from that found in ferret, where OFF cells fire more often than ON cells [21],[22].

Here, we report results from a modeling study of the properties of experimentally-proposed synaptic plasticity rules and modifications to these rules necessary to capture the segregation of ON and OFF retinal inputs to a postsynaptic LGN neuron driven by recorded RGC spike trains [20]. We take two approaches to investigate this problem: (i) by making various assumptions, we reduce the system to one that is analytically tractable, to allow us to use eigenvalue theory to predict synaptic weight development [22]–[24]; (ii) computational simulations allow us to test a larger system with less-restrictive assumptions. Combining these approaches gives us insight into why the models perform as they do. Unsurprisingly, we find that a naively-implemented (additive) pair-based STDP [25] cannot segregate ON and OFF inputs using experimental values for the balance between synaptic potentiation and depression [9],[10],[26]. Since STDP integrates spikes over tens of milliseconds, on this timescale ON/OFF correlations are much smaller than ON/ON and OFF/OFF correlations, and both ON and OFF RGCs successfully drive the postsynaptic neuron leading to potentiation of both cell types. Therefore, homeostatic mechanisms must be implemented to induce synaptic weight competition [27],[28]. However, for biological ratios of depression to potentiation close to unity, the segregation outcome is highly sensitive to the choice of parameters. This sensitivity aries since STDP with approximately balanced potentiation and depression does not utilize the correlations between cells but rather the cells' time-averaged firing rates. Within the millisecond-long integration timescales, the second-long RGC correlations appear noisy and essentially constant. Due to the asymmetry of STDP potentiation and depression effects cancel each other, such that the signaling component of STDP for segregation is diminished. Since longer integration timescales are more appropriate for capturing the firing patterns of the two input types, when we extend these timescales of STDP to half a second, segregation is always biased towards the cell that fires first, ON. We find that BTDP can robustly drive ON/OFF segregation in the LGN without requiring normalization, because in addition to the second-long timescales, it integrates pre- and postsynaptic activity irrespective of temporal order. The segregation outcome results from a trade-off between the shorter high-frequency bursts of ON cells, and the more strongly correlated OFF cells. Despite differences in firing patterns, our model can also reproduce ON/OFF segregation in ferret [22], suggesting a universality of the rule which governs ON/OFF segregation across species.

## Results

**Figure 1:**
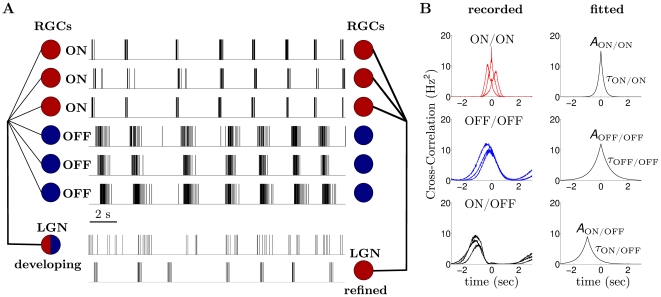
Model description and input correlation functions. (A, left) An LGN neuron receives feedforward weak synaptic input from neighboring ON (red) and OFF (blue) RGC inputs early in development. (A, right) Spontaneous retinal waves selectively refine RGC inputs, such that synaptic weights of one RGC type (ON) strengthen, while weights of the other RGC type (OFF) decay to 0, resulting in an ON-responsive LGN neuron. Sample P12 spike rasters in the middle demonstrate that ON cells fire shorter bursts of higher spike frequency, while OFF cells fire longer bursts of lower spike frequency, 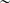
1 second after ON [20]. Also shown are spike rasters for a developing LGN neuron receiving weak mixed ON and OFF inputs, and for a refined LGN neuron, receiving selective input solely from ON RGCs. LGN spiking activity was generated using the integrate-and-fire model in Equations 12–13. (B) Correlation functions for the input spike trains shown in (A) (mouse data set 1). The input correlation function for RGCs of the same type (ON/ON and OFF/OFF) peaks at 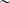
0 seconds, while for RGCs of different type peaks at 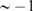 second for ON/OFF and 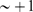 second for OFF/ON pairs. Symmetric decaying exponentials were fit to the pairwise input correlations using Equation 11. Peak amplitudes and decay time constants for pairs with maximal peaks are reported in Table 1.

**Figure 2:**
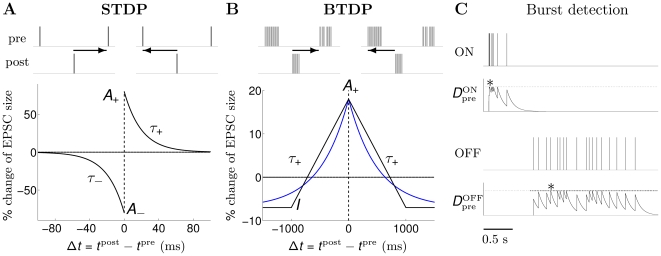
STDP versus BTDP and burst detection. (A) STDP modifies synaptic strength based on the timing, 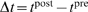 (indicated by horizontal arrows), and firing order of pairs of pre- and postsynaptic spikes [10]. Synaptic change occurs in a time window on the order of tens of milliseconds determined by 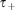 and 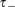, with maximum change at 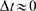 given by 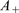 and 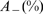 in EPSC (evoked postsynaptic current) size. (B) BTDP governs synaptic change based on the timing (but not order) of pre- and postsynaptic bursts over a second-long time window, 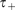 [16]. We fit the blue symmetric exponential curve to the linear experimental fit, where 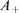 and 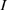 denote the amounts of maximum synaptic potentiation and depression in 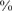 of EPSC size, respectively. (C) We detected ON (top) and OFF (bottom) bursts by accumulating burst detection variables 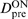 and 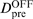. At the arrival of a spike, 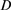 increases by 1, and otherwise decays exponentially with a time constant 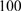 ms. A burst was detected once 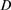 reached a fixed threshold (here 1.5) denoted with an asterisk; 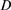 was not allowed to exceed the threshold value. The location of the asterisk (instead of the start time of the burst) was used to evaluate the burst latency for synaptic change. Parameters for STDP and BTDP are listed in Table 3. Figure modified from [68] with permission from the HFSP journal.

In Figure 1A we illustrate the model used in this study, consisting of multiple ON and OFF RGC inputs projecting in a feedforward manner to a postsynaptic neuron in the LGN. Inputs to the model were experimentally-recorded activity patterns in the form of spike trains from P12 mouse retina by Kerschensteiner and Wong [20], with 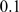 ms precision and duration of 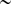
1 hour. Six experimental data sets were used (all recordings available online as Dataset S1), each consisting of 5–8 mixed (ON and OFF) RGC inputs recorded by one field of the multielectrode array used by Kerschensteiner and Wong [20]. Due to the high density of electrodes within one recording field (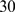 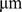 between electrodes), differences in distance-dependent correlation among RGCs within the same data set were not relevant at this age [29]. RGCs were previously identified by Kerschensteiner and Wong as ON or OFF by light modulation; at the age studied, 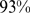 of the total recorded RGCs showed a response exclusively to light onset or offset [20]. This suggests that relatively few RGCs at this stage display mixed ON and OFF responses. We report input statistics (firing rates, numbers of ON and OFF inputs in each data set) in Table 1. Given these inputs, the activity of the postsynaptic LGN neuron was generated using two different models (Materials and Methods): in a biologically-realistic model we simulated the postsynaptic neuron with nonlinear integrate-and-fire dynamics and used as inputs the spike trains of all ON and OFF RGCs in each data set; in a reduced model we based postsynaptic activity on a linear Poisson neuron, and derived a linear equation for the dynamics of two synaptic weights, one ON and one OFF, using input statistics fits of the most correlated ON/ON and OFF/OFF cell pairs (Figure 1B). Change in synaptic strength was governed by STDP (Figure 2A) or BTDP (Figure 2B), where Figure 2C illustrates how bursts were detected. The two modeling approaches (linear Poisson and nonlinear integrate-and-fire models) complement each other in identifying which plasticity rule can read out key retinal wave properties for ON/OFF segregation.

**Table 1:** Peak amplitudes and decay time constants of the symmetric fall-off exponential fits to the correlation functions of the most-correlated input pair from spontaneous retinal wave recordings by Kerschensteiner and Wong [20] (values given as estimate
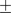
standard error). 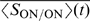 and 
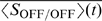
 denote the average firing rates of the two most correlated ON and OFF cells in each data set; other parameters are illustrated in Figure 1B.

Set		1	2	3	4	5	6
# ON cells		3	6	3	5	4	3
# OFF cells		3	2	2	2	2	5
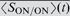	(Hz)	1.51	0.40	0.70	1.01	0.34	1.04
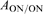	(Hz^2^)	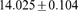	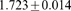	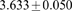	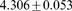	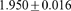	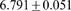
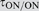	(s)	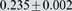	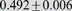	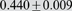	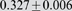	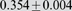	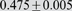
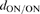	(s)	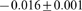	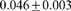	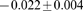	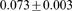	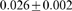	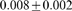
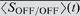	(Hz)	2.94	0.63	0.80	2.44	2.06	2.48
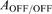	(Hz^2^)	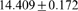	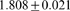	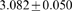	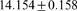	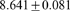	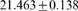
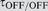	(s)	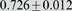	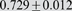	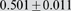	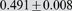	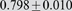	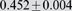
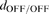	(s)	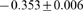	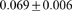	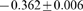	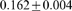	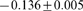	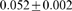
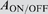	(Hz^2^)	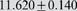	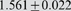	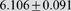	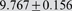	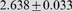	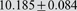
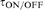	(s)	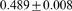	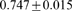	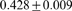	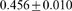	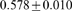	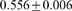
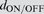	(s)	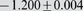	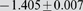	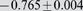	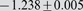	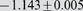	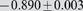

The number of ON and OFF cells used in the simulated integrate-and-fire model are also listed for each data set. The OFF/ON correlation function is a reflection about 0 of the ON/OFF correlation function, such that 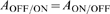, 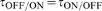 and 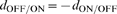.

### Standard STDP with 20 ms timescales for spike integration fails to guide ON/OFF segregation in realistic parameter regimes

First, we studied a standard pair-based STDP rule with additive synaptic change (Figure 2A) [25] under the reduced linear model framework (Materials and Methods) [24]. Comparing typical input correlation timescales used by the linear model (for example, data set 1 in Figure 1B) with the timescales of spike integration in STDP (Figure 2A), suggested that the resulting weight dynamics would be determined by the relative strength of the correlation between ON/ON and OFF/OFF pairs for a small time lag around 0 ms (Figure 1B, top and middle panels), because the millisecond-long correlation window of STDP would ignore the 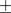
1 second offset in the ON/OFF and OFF/ON correlation pairs (Figure 1B, bottom panel). We explored segregation outcome as a function of the balance between depression and potentiation effects in the plasticity rule, denoted by the ratio of the negative and positive integrals of STDP, 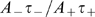 (Figure 2A). Most experimental data have reported similar integration timescales for depression and potentiation, and approximately equal amplitudes [30], or even a slight dominance of potentiation, thus, suggesting a depression-to-potentiation ratio less than or equal to 1 (for example, 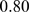 [10], and 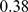 [9]). Under these limitations, we studied STDP with equal potentiation and depression timescales of 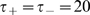 ms, which simplifies the depression-to-potentiation ratio to the ratio between amplitudes, 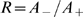. Fixing the maximum potentiation amplitude 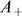, we varied 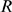 through the maximum depression amplitude 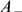.

**Figure 3:**
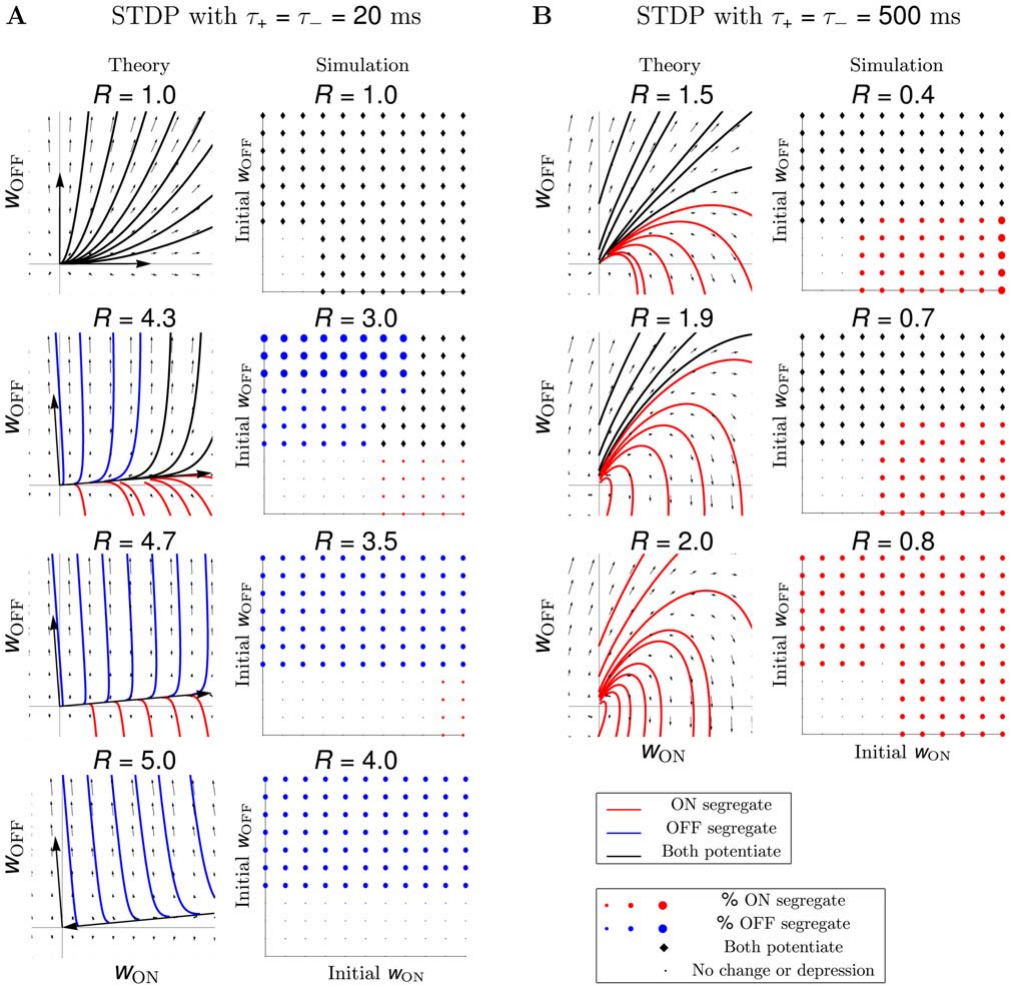
STDP without normalization does not result in segregation. (A) STDP with spike integration time windows of 20 ms results in ON/OFF segregation only for two data sets out of six (data set 1 shown here; data set 2 not shown), assuming a depression-to-potentiation ratio, 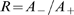, much larger than experimentally-observed. Theory and simulation show similar trends, although actual values of 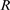 differ. (Theory) Vector fields and example trajectories of the ON-OFF weight dynamics illustrate results from the linear model. The direction of two eigenvectors of the plasticity matrix 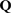 (pointing out of the origin for a positive eigenvalue, and into the origin for a negative eigenvalue) determine how 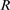 affects segregation: as 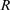 increases, regions of initial conditions where both weights potentiate (trajectories in black) become areas where segregation occurs (trajectories in red for ON and blue for OFF). (Simulation) In the simulated integrate-and-fire model (here, data set 1 with 3 ON and 3 OFF inputs), a separate simulation was run for each initial condition uniformly sampled between 0 and the maximum weight value, 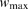, for the ON and the OFF weights. Here 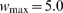 and the discrete steps for the initial weights were 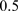. The colored symbol indicates the segregation outcome according to the legend, matching the color of the trajectories in the linear model. The size of the colored circles denotes the percentage of synaptic weights of a particular RGC type which potentiated maximally out of all RGCs of the same type initially wired to the LGN neuron (the three dots denote 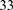, 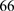 and 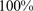, respectively), while all weights of the other RGC type depressed to 0. Initial weights too small to generate postsynaptic activity result in no synaptic change (dots in the bottom left region of each plot). (B) Extending the spike integration window to match the timescale of the input correlations to 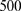 ms, results in pure dominance of the ON cells, both under the theoretical and the simulated model and for any value of the ratio 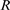 (data set 1 shown here, but all others show the same behavior).

In Figure 3A (theory) we show the weight dynamics for data set 1 for different values of 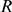. The vector fields and example trajectories demonstrate the segregation outcome for any initial condition in the range 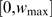 for the ON and OFF weights. For experimentally-observed ratios of 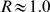, any initial condition led to the maximal weight potentiation of both cell types (top panel). Increasing 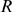 replaced the region where all weights grow, with regions where depending on initial conditions either ON or OFF segregation occurred (two middle panels). Many initial condition combinations (even those giving a prominent bias to ON) resulted in OFF segregation (bottom panel). We confirmed segregation results from the reduced linear model by simulating spikes for the postsynaptic LGN neuron with an integrate-and-fire model in Figure 3A (simulation), where all ON (3) and OFF (3) spike trains from data set 1 were used as inputs. The space of all initial conditions for the ON and OFF weights in 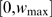 was explored in discrete steps, such that a separate simulation was run for each combination of initial conditions. Both theory and simulation demonstrated a similar trend for increasing 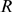 (corresponding panels in Figure 3A, theory and simulation), though not surprisingly the ratios did not closely match.

**Figure 4:**
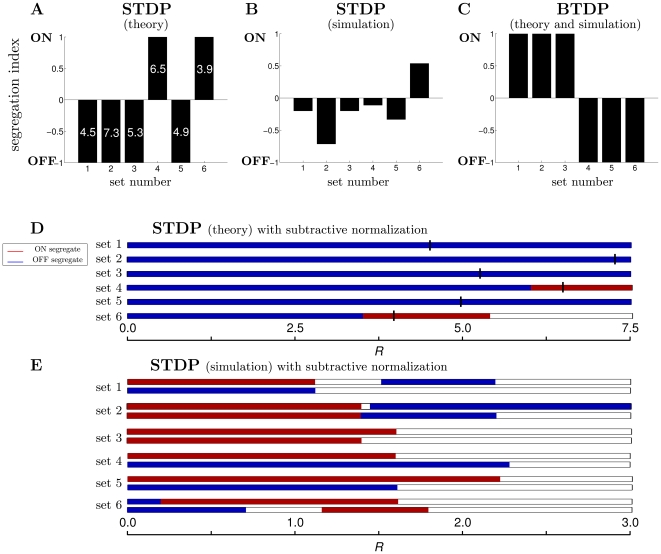
A summary of segregation under STDP and BTDP. (A) Segregation indices using Equation 16 for all data sets following the linear modeling approach with STDP using unbiased initial conditions of 4.0 for both ON and OFF weights. By design the theoretical model always results in segregation for large enough 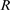; the minimum 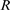 for segregation is written in each bar. (B) Segregation indices from numerically implementing STDP for all data sets illustrate the absence of segregation. A high 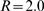 was used here; increasing 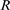 beyond 3.0 results in segregation only for data sets 1 (Figure 2A) and 2 (data not shown), inconsistent with linear model predictions in (A). (C) Segregation indices from the theoretical and numerical implementation of BTDP show consistent segregation across all data sets using the experimentally-observed ratio 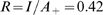 [16]. (D) STDP with subtractive normalization can induce weight competition and segregation in the linear model. For realistic depression-to-potentiation values of 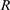, the outcome is always OFF segregation. Increasing 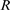 to the values which generated segregation in (A) (denoted by the black vertical lines) results in ON segregation for sets 4 and 6, matching results from (A). Note that in the linear model 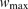 simply scales the weights and thus, it does not affect segregation outcome. (E) STDP with subtractive normalization also results in segregation in the simulated integrate-and-fire model for realistic values of 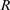. However, results depend on parameters: for instance, changes in 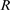 (horizontal axis) sometimes result in a switch from ON to OFF segregation (data set 2), and sometimes from ON to no segregation (data set 3). Also, the amount of total synaptic weight maintained by the postsynaptic neuron (top bar for each set 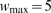 and bottom bar 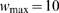) affects segregation outcome (for example, data sets 4 and 5). While subtractive normalization rescues segregation for the STDP rule, results are inconsistent across the two different modeling approaches (compare to D). Uncolored sections in each bar denote no segregation.

Comparing results from the reduced linear Poisson model and the simulated integrate-and-fire model for the other data sets (data not shown), however, did not produce consistent results as for data set 1. To simultaneously demonstrate the difference in outcomes between the two models, and the absence of segregation across all data sets, we computed a segregation index (Equation 16, Materials and Methods). While a detailed presentation of the segregation results using each modeling approach and for the whole range of initial conditions in 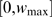 can be made for all data sets as for data set 1 in Figure 3A, for the sake of brevity we present only the segregation index with fixed unbiased initial weights of 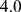 for both ON and OFF in Figure 4A,B. The segregation index was, in fact, the same for all unbiased initial conditions (uniformly distributed along the main diagonal of the two-dimensional weight space) for all data sets as for data set 1 in Figure 3A. The reduced linear model is two-dimensional, thus, by design segregation occurs when one weight wins and the other loses; this always happens if 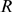 is sufficiently large. Figure 4A shows the segregation index under the reduced linear model framework, where the numbers in each bar denote the minimum 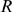 required for the selected unbiased initial conditions to give segregation. A common requirement for segregation across all the data sets was a depression-to-potentiation ratio 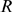 much larger than observed experimentally [9],[10], and in theoretical work with synthetically-generated Poisson inputs [24],[25]. This was needed to account for the large pairwise correlations of the real spike recordings, in contrast to the small cross-correlations between independent Poisson inputs which need 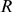 slightly larger than one (for example, 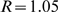) [25].

Unlike the reduced linear model, segregation in simulations with the integrate-and-fire model is harder to achieve, since it requires a subset of all weights of one cell type to potentiate maximally, while all weights of the other cell type to depress. Only in data sets 1 (Figure 3A) and 2 (data not shown) we found that increasing 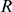 beyond 3.0 resulted in segregation for a range of initial conditions, consistent with the reduced linear model in Figure 4A. Segregation was not achieved in the other data sets for any studied 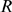. Though it is possible that we did not sample the range of 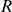 finely enough, a value of 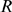 which could lead to segregation would require careful tuning because of two observations: (i) increasing 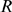 beyond some value eventually led to depression of all weights, and (ii) for smaller values of 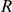 for which we did observe potentiation of some weights, the result was always potentiation of both ON and OFF weights as these tightly correlated RGCs of different cell type reliably drove postsynaptic spikes within 5–10 ms. A summary of the segregation index simultaneously showing the absence of segregation for all data sets is given in Figure 4B for a sample ratio of 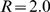 and unbiased initial conditions of 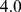 for both ON and OFF weights.

We also explored an STDP rule with a longer temporal window for synaptic depression than for potentiation corresponding to experiments from somatosensory cortex, 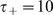 ms and 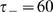 ms [26]. Now the ratio of the negative to positive areas of the STDP integral, 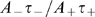, took larger values by considering smaller ratios of the depression and potentiation amplitudes, 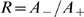. However, only data set 1 resulted in OFF segregation for this choice of STDP parameters (data not shown).

In conclusion, our modeling showed that a temporally-asymmetric plasticity rule like STDP, integrating pre- and postsynaptic spikes over short timescales on the order of tens of milliseconds which ignore the correlation timescales between inputs of different cell type, failed to segregate these ON and OFF RGC inputs in a model of a developing LGN over a wide range of parameters and in the absence of synaptic competition. Under STDP, the growth of synaptic weights of one cell type did not prevent the growth of weights of the other cell type, because ON and OFF cells fire independently within the timescale of STDP. Therefore, tightly correlated groups of ON and OFF cells which effectively drive the postsynaptic neuron, required physiologically-unrealistic, carefully-tuned values of the depression-to-potentiation ratio, 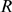, for segregation. In the integrate-and-fire model even large 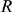 could not rescue segregation, because multiple ON and OFF inputs caused reliable spiking of the LGN neuron, and large 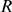 resulted in nonselective competition between all ON and OFF cells, rather than between groups of ON and OFF cells. These results are not too surprising given recent theoretical work on STDP [31]. Introducing dependence of weight change on the current weight, choosing a different spike-pairing scheme or implementing dendritic and axonal synaptic delays may differently affect the behavior of the modeled system [31]. Furthermore, existing correlation-based models of ocular dominance require some form of synaptic competition to segregate inputs from the left and the right eye [32], which we considered next.

### STDP with homeostatic mechanisms can rescue segregation in carefully-tuned regimes

It is likely that synaptic plasticity rules work together with homeostatic mechanisms during formation and refinement of developing circuits [33]. While most work has examined developing cortical neurons where synaptic scaling is induced in response to changes in global activity levels [34],[35], there is recent evidence of response homeostasis at the developing retinocollicular system maintained at the level of synapses [36]. We considered the implications of this second type of homeostasis together with STDP on segregation. Chandrasekaran et al. [36] found that at the level of a single postsynaptic neuron in the superior colliculus, the total number and strength of individual synapses is preserved during development. We implemented this as subtractive normalization, because it has been shown to induce weight competition (in contrast to divisive normalization) in traditional forms of Hebbian learning [27],[37]. For each data set, we chose a number of synaptic weights 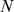 that the postsynaptic LGN neuron would maintain at adulthood with maximal strength 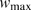, such that the total synaptic input to the LGN neuron maintained at all time during the simulation was 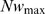. In addition to STDP, synaptic weights were modified by adding/subtracting the synaptic deficit/excess towards maintaining the target synaptic strength (Materials and Methods). As in STDP with large 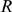 (Figure 4A), STDP with subtractive normalization for the reduced linear model where a single ON and a single OFF weight competed, also resulted in segregation but for biologically-plausible values of 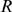 (Figure 4D). However, the segregation outcome for 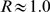 in Figure 4D did not match results from STDP without normalization in Figure 4A, and in particular the outcome was always OFF segregation (Table 2). While same cell type correlations are higher than opposite cell type correlations, when considered within the millisecond-long window of STDP these second-long correlations are noisy and essentially constant. Then for biologically-plausible ratios of depression-to-potentation near unity (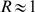) the effect of the correlations is diminished, because the contributions from the potentiating and from the depressing part of temporally-asymmetric STDP cancel. In this case, the evolution of the weights is entirely determined by single neuron properties, and in particular, by the time-averaged firing rate of each cell. The OFF cells for all data sets have larger firing rates than the ON cells (Table 1), leading to a bias for OFF segregation in Figure 4D. As 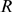 increases, the correlations start to become more relevant. The product of the correlations with STDP in Equation 8 (Materials and Methods) is significantly negative for large 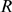 for all cell pairs, and moreover, it is more negative for same cell types due to their larger correlations in comparison to opposite cell types. Therefore, for some data sets where the negative contribution of the correlations dominated the positive contribution of the average firing rates (data sets 4 and 6), the ON cell with lower firing rate won for sufficiently large 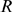. In particular, for 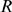 equal to the values which resulted in segregation without normalization in Figure 4A (indicated by the short vertical lines in Figure 4D), data sets 4 and 6 produced ON segregation consistent with Figure 4A.

**Table 2:** Parameter sensitivity of STDP with subtractive normalization.

Model	set 1	set 2	set 3	set 4	set 5	set 6
**2D linear**	OFF	OFF	OFF	OFF	OFF	OFF
**full linear**	OFF	OFF	—	OFF	OFF	OFF
**I&F with** 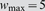	ON	ON	ON	ON	ON	ON
**I&F with** 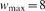	OFF	ON	ON	ON	OFF	ON
**I&F with** 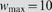	OFF	ON	ON	OFF	OFF	—
**I&F with** 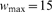	OFF	OFF	ON	OFF	OFF	—
**I&F with** 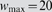	—	OFF	—	OFF	OFF	—

Segregation results are shown for the reduced two-dimensional linear model, the full linear model with all RGC inputs, and the nonlinear integrate-and-fire model with all RGC inputs for a biologically-plausible range of the depression-to-potentiation ratio 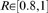. ON denotes ON segregation, OFF denotes OFF segregation, and a dash denotes no segregation.

In the nonlinear integrate-and-fire model where multiple ON and OFF inputs were used, implementation of homeostasis also rescued segregation for biologically-plausible values of 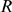 (Figure 4E). However, even with subtractive normalization the final outcome in simulations depended on parameter choice (depression-to-potentiation ratio 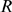 and maximum synaptic strength 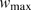) and differed from the outcome of the reduced linear model (Figure 4D,E and Table 2). To understand whether the dependence of the segregation outcome was the result of variable input statistics, or the nonlinearity of the integrate-and-fire model, we simulated the full linear model using all RGCs in each data set. In Table 2 we summarize the segregation outcome for a realistic depression-to-potentiation ratio 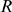 between 0.8 and 1.0, under the three different models: the reduced linear model (Figure 4D), the full linear model, and the integrate-and-fire model, for a range of different 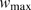 values (Figure 4E). Both the reduced and the full linear model had a preference for OFF segregation for all data sets. Above, we explained that the reason for this OFF bias is due to the diminishing effect of the correlations under STDP and the dominance of firing rates (which are generally higher for the OFF cells) for 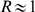. As an exception, the full linear model for data set 3 failed to capture OFF segregation because the multiple ON and OFF cells had more variable firing rates than the consistently larger OFF firing rates in the other data sets. With the output nonlinearity of the integrate-and-fire model, we observed two regimes: (1) for a small 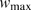, the ON cells won in all data sets (Table 2) unlike the linear model, probably because the short timescales of STDP captured the high instantaneous firing rates of ON cells within a burst (as indicated by the smaller decay timescales for ON/ON than OFF/OFF RGC pair correlations in Table 1). The OFF cells most likely failed to drive the postsynaptic neuron as efficiently as the ON cells, due their lower instantaneous firing rates within a burst; (2) for a large 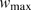, the OFF cells won in most data sets in agreement with the linear model. The exact transition point from (1) to (2) depended on the data set (Table 2). Thus, fine-tuning of 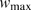 is necessary to develop similar numbers of ON-selective and OFF-selective LGN neurons. Again, the sensitivity to 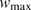 arises because STDP does not exploit the correlations between cells, but rather the time-averaged firing rates. In the regime of 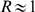, the contributions of the potentiation and depression regions of STDP cancel with each other yielding dominance of the time-averaged firing rates and OFF segregation. In the less-relevant regime of 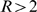, STDP suppresses cooperation between same type cells. While in the reduced linear model this increase in 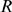 resulted in a switch from OFF to ON segregation for data sets 4 and 6, in the full linear model and in the simulated integrate-and-fire model, it introduced stronger competition between same type than between opposite type RGCs, eventually eliminating segregation (Figure 4E, large R).

In summary, we showed that adding subtractive normalization to STDP resulted in ON/OFF segregation but the outcome was highly sensitive to the choice of parameters. For biologically-realistic depression-to-potentiation values of 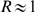, temporally-asymmetric STDP with normalization consistently favored one cell type over the other, depending on the model of the postsynaptic neuron. We determined a parameter range for 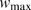 where the linear model can approximate the nonlinear model, but to obtain an equal preference for ON or OFF segregation, fine-tuning of 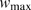 was critical. Even though it is plausible that the sensitivity of the model to various parameters may be implemented by a biological system during development, the model predicts that the system will produce very different outcomes for any small perturbation induced by environmental changes, stochastic events, unreliable vesicle release, and so on, which seems unlikely. The millisecond-long integration timescales of STDP are too short to reliably sample the noisy correlation functions which naturally extend over much longer timescales, thus making STDP unsuitable to study segregation in this system. Later in the paper we explore a more appropriate plasticity rule modulating synaptic change and during development, which resulted in segregation without the need for presynaptic homeostatic control or careful parameter tuning (results summarized in Figure 4C).

### Temporally-asymmetric STDP with second-long timescales favors ON segregation

A spike-based rule like STDP cannot explain ON/OFF segregation in the LGN without synaptic competition because tightly correlated retinal inputs of different cell type reliably drive postsynaptic activity within 5–10 ms. Even with subtractive normalization, the short timescales of STDP are ineffective at robustly driving ON/OFF segregation. Butts and Rokhsar [14] found that most information content of spontaneous retinal waves is contained over timescales of 100–1000 ms. Furthermore, Butts et al. [16] recently proposed a burst-based rule for modifying synaptic strength over second-long timescales in the developing retinogeniculate system. We next asked if *any* plasticity rule integrating pre- and postsynaptic activity over the relevant input correlation timescales of ON and OFF RGCs can explain segregation. Thus, we investigated a second plasticity rule, a modified STDP with extended spike integration timescales.

Vector fields and example trajectories in Figure 3B (theory) illustrate segregation scenarios produced with the reduced linear model for data set 1 with 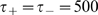 ms in STDP. We confirmed these in Figure 3B (simulation) with the integrate-and-fire model where all ON (3) and OFF (3) inputs in data set 1 were used with multiple (discretely-sampled) initial conditions in 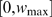 for the ON and OFF weights. As the depression-to-potentiation ratio 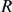 increased, the region of initial conditions for which weights of both cell types potentiated was gradually replaced with a region of ON segregation. Segregation results followed a similar qualitative trend for the other five data sets (data not shown). In conclusion, modifying synaptic weights based on STDP with second-long timescales for spike integration to match input correlation timescales resulted in ON segregation for all data sets and for any combination of initial conditions. This effect was not only produced by the reduced linear model, but also by the simulated integrate-and-fire model for the LGN neuron. Despite different numbers of ON and OFF retinal inputs in each data set (Table 1), a subset of ON synaptic weights always potentiated, while all OFF weights were eliminated. Since LGN neurons can be both either ON- or OFF-responsive [17],[38],[39], we conclude that such a rule is implausible as it never results in OFF-responsive LGN neurons.

These modeling studies demonstrate that a plasticity rule for segregation does not only have to integrate pre- and postsynaptic activity over timescales matching those of the input correlations, but that other constraints are also required. Since the firing of ON RGCs precedes that of OFF RGCs, any rule which integrates activity over the second-long correlation timescales, must do so without giving a naïve advantage to the cell which fires first. STDP, on the contrary, favors synaptic inputs that can serve as ‘earliest predictors’ of other spike events [25],[40]. Now we turn to BTDP which integrates pre- and postsynaptic bursts irrespective of their firing order.

### Temporally-symmetric BTDP with second-long timescales robustly guides ON/OFF segregation

**Figure 5:**
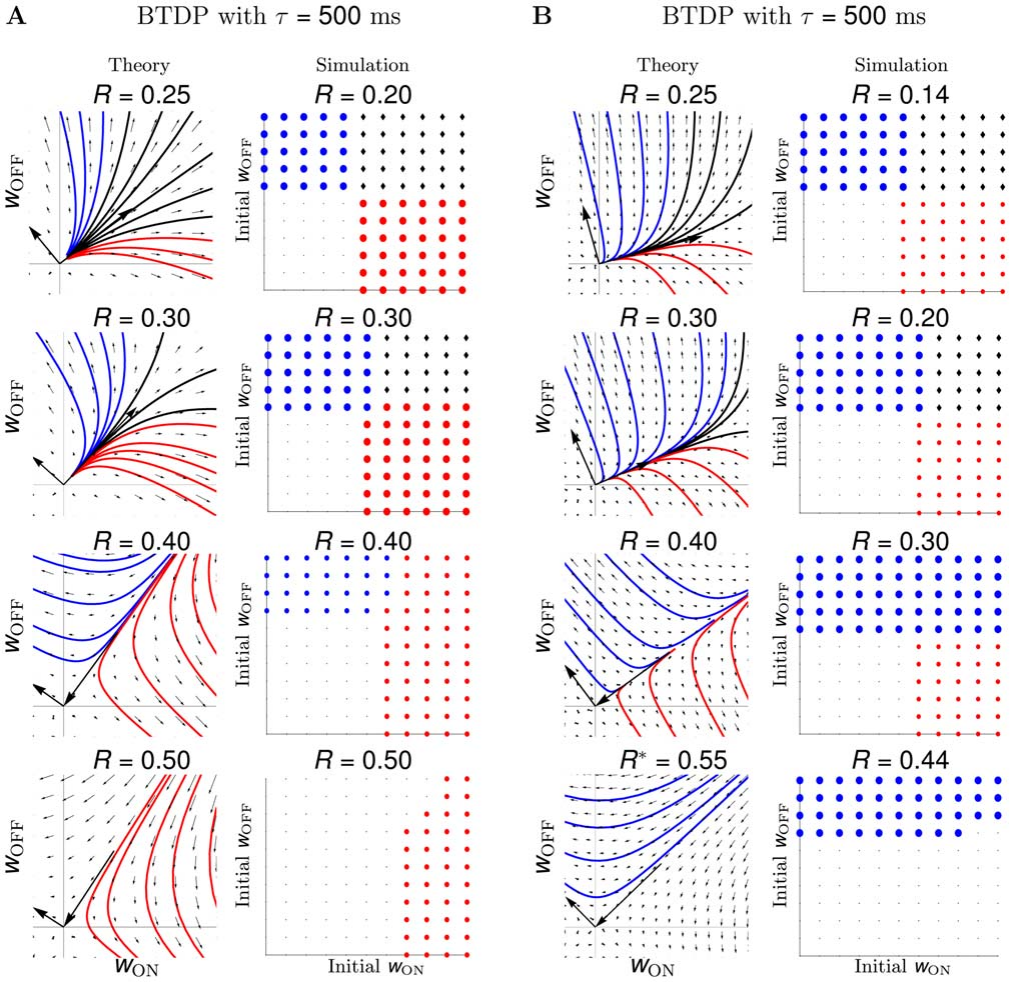
BTDP results in robust segregation. (A) Segregation results using BTDP for data set 1, a representative spike train recording of data sets 1–3 which have similar peaks of the pairwise input correlation for ON/ON and OFF/OFF pairs. Drawing conventions and legend as in Figure 2, and as before 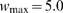 with discrete steps for the initial weights of 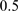. As the depression-to-potentiation ratio in BTDP, 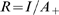, increases towards the experimentally-observed ratio, 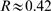 [16], theory and simulation show the segregation of ON and OFF RGC inputs (red and blue trajectories), emerging from a state where the weights of both cell types potentiate maximally for small 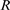 (black trajectories). The region of initial conditions resulting in ON segregation is larger than the region resulting in OFF segregation, indicative of ON dominance (see main text). Similarly, unbiased initial conditions located along the main diagonal in each plot show ON segregation. (B) Segregation results using BTDP for data set 4, a representative spike train recording of data sets 4–6, which have higher OFF/OFF input correlation peaks than ON/ON peaks. As 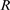 increases, the dominant segregation outcome for a larger set of initial conditions is OFF segregation, in contrast to (A). While the match between theory and simulation is consistent for data sets 4–6 and experimentally-observed 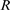 [16], for data sets 4 and 5 a narrow range of large ratios (denoted by 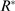) resulted in OFF segregation only in simulations (bottom panels). To match predictions of the two models for this larger than experimentally-observed 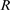, 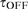 was made larger than 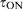 in the theoretical model (from 0.327 to 0.500, Table 1).

Can a temporally-symmetric burst-based rule integrating pre- and postsynaptic bursts over second-long timescales guide ON/OFF segregation in the LGN? Such a rule, BTDP, was experimentally-proposed for the developing retinogeniculate system and tested in a model for eye-specific segregation using simulated retinal waves [16]. We found that BTDP robustly guides ON/OFF segregation in all data sets (Figure 5), without requiring homeostatic control of presynaptic connectivity to introduce synaptic competition. BTDP resulted in segregation assuming a realistic, experimentally-observed ratio of depression-to-potentiation 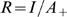 (Figure 2B). (Although R is defined differently for STDP and BTDP, it has the same meaning in both rules.) Segregation outcome fell under two qualitative trends: dominance of ON segregation (data sets 1–3 in Dataset S1, Figure 5A) and dominance of OFF segregation (data sets 4–6 in Dataset S1, Figure 5B), where ‘dominance’ was defined as which cell type normally won, averaged over all initial conditions.

In Figure 5A (theory) we show the segregation outcome under the reduced linear model which used as inputs the correlation functions (Table 1 and Figure 1B) for data set 1, as a representative of data sets 1–3. Fixing the symmetric timescale of integration 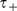 and the maximum amplitude of potentiation 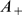 according to Table 3, we varied the depression-to-potentiation ratio 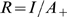 by changing the amount of depression 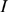. As 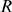 increased towards the experimentally-observed value of 0.42 [16], initial conditions which originally led to the maximal potentiation of both weights (top panel), now resulted in ON or OFF segregation (two middle panels). ON segregation was the only outcome for large 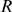 (bottom panel). Further increasing 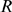 led to overall depression of all weights (data not shown). The results with the reduced linear model driven by the correlation functions of the most correlated ON and OFF cell pairs were confirmed when simulating spiking activity using a nonlinear integrate-and-fire neuron for the LGN neuron driven by all ON (3) and all OFF (3) spikes trains in data set 1 (Figure 5A, simulation). For each combination of initial conditions of ON and OFF weights and experimentally-observed ratio 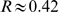 [16], some weights of only one cell type potentiated, while all weights of the other type depressed, indicating successful segregation (Figure 5A, simulation, third panel). Since there were different numbers of ON and OFF inputs in each data set (Table 1), our results show that segregation is robust and is not biased by the initial numbers of ON and OFF RGCs connecting to the LGN neuron. Furthermore, the match between any 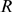 of the reduced linear and the simulated integrate-and-fire model was remarkably good. We called the trend illustrated in Figure 5A dominance of ON segregation, because for the experimentally-observed ratio 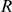 [16], more initial condition combinations resulted in ON segregation than in OFF segregation (Figure 5A, third panels). In particular, unbiased initial conditions for the ON and OFF weights (located along the main diagonal), and even a small OFF bias in initial conditions, resulted in ON segregation. Data sets 2 and 3 showed qualitatively similar dominance of ON segregation (data not shown).

**Table 3:** Model parameters for generation of LGN activity using the linear model (theory) and the integrate-and-fire model (simulation), and for synaptic modification induced by STDP or BTDP.

Parameter	Notation	Value
**Theory**		
EPSP kernel 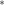	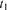 (ms)	10
		100
	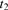 (ms)	5
		50
STDP/BTDP	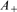	0.001
**Simulation**		
Integrate-and-fire	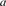	0.02
	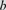	0.2
	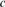	−50
	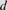	2
	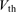	30
STDP/BTDP 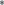	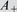	0.0005
		0.0001
**Common parameters**		
Upper synaptic bound 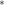	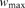	5
		10
		20
STDP 	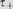 (ms)	20
		10
		500
	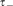 (ms)	20
		60
		500
BTDP 	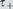 (ms)	500

Multiple values were tested for some parameters, marked by asterisks.

The segregation outcome under the reduced linear model for data set 4, as a representative of data sets 4–6, is illustrated in Figure 5B (theory). As before, we increased 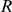 towards the experimentally-observed value of 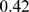 [16] and saw that initial conditions which led to the maximal potentiation of both weights for small 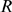 (top panel), now resulted in ON or OFF segregation (two middle panels). To obtain OFF segregation as the only outcome for 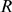 larger than experimentally-observed in the same way we obtained ON segregation for data sets 1–3 in Figure 5A (bottom panels), we had to extend the correlation timescale (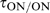) of ON RGC pairs in Figure 5B (theory, bottom panel). Simulating spiking activity using a nonlinear integrate-and-fire model for the LGN neuron driven by all ON (5) and all OFF (2) spike trains in data set 4 matched the segregation results from the reduced linear model, with a close correspondence of 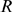 (Figure 5B, simulation). Later we discuss why for large 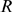 the simulated model, but not the theoretical model, resulted in OFF segregation for all initial conditions. As before, segregation was robust and did not depend on the initial numbers of ON and OFF retinal inputs (Table 1). Data sets 4–6 showed dominance of OFF segregation, for the experimentally-observed ratio 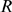 [16] shown in Figure 5B (third panels). Furthermore, unbiased initial conditions for the ON and OFF weights (located along the main diagonal), and even a small ON bias in initial conditions, resulted in OFF segregation. Data sets 5 and 6 showed qualitatively similar dominance of OFF segregation (data not shown).

To summarize, we found that not only the second-long timescales of bursts integration are needed for segregation, but also the temporally-symmetric feature of BTDP. In particular, the negative and symmetric BTDP window at long temporal delays in firing, mutually inhibited the simultaneous growth of ON and OFF synaptic weights, thus providing the necessary synaptic competition without implementing additional homeostatic mechanisms, in contrast to STDP. In Figure 4C, we summarize the segregation results of BTDP for all the data sets using experimental values of 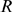 [16], and unbiased initial conditions. Figures 4C and 5A,B demonstrate that the structure of the inputs, in combination with initial conditions for the ON and OFF weights, determine the segregation outcome such that data sets 1–3 showed dominance of ON segregation, while data sets 4–6 dominance of OFF segregation. Mathematical interpretation of the result based on this simple model is explored later in this paper.

### Upper bound for synaptic weights does not affect segregation under BTDP

**Figure 6:**
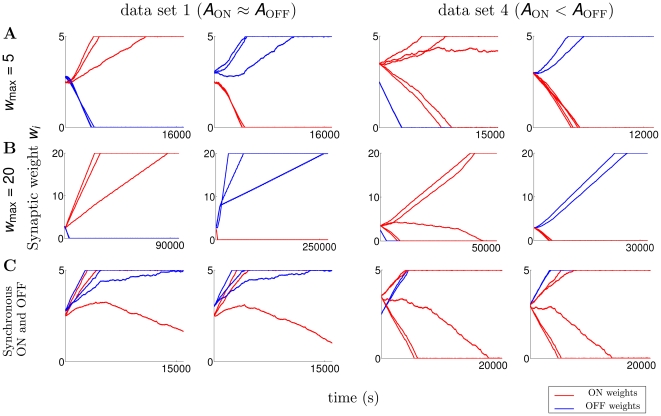
Features which guide segregation under BTDP. (A) Temporal evolution of synaptic weights with 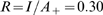 for data set 1 (3 ON and 3 OFF cells), as in Figure 4A, for initial conditions when the ON (first column) or the OFF cells (second column) segregate. Similarly, sample trajectories for data set 4 (5 ON and 2 OFF cells), as in Figure 4B, for initial conditions where the ON (third column) or the OFF cells (fourth column) segregate. Upper bound on the weights 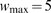. Even though there are more ON than OFF inputs in data set 4, the outcome does not depend on the number of ON and OFF inputs, but on the initial conditions of the synaptic weights. Furthermore, a simple bias in the initial conditions does not always result in segregation towards the biased cell (see Figure 4 for the segregation outcome for all initial condition combinations). (B) Increasing the upper bound of the weights to 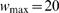 does not affect the segregation outcome; weights simply take longer to segregate. (C) Delaying the time of the spikes of ON cells by 1 second, such that they are synchronous with OFF cells, eliminates segregation.

The ratio of final (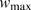) to initial synaptic strength of the winning cell type after segregation indicates the total amount of synaptic strengthening during this stage of development. Chen and Regehr [6] showed that in addition to the pruning of RGC inputs converging to a single LGN neuron (from 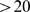 inputs at birth, to 1–3 after eye-opening), synaptic weights strengthen 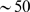-fold. According to more recent estimates, 5–8 input RGCs before the onset of glutamatergic waves refine to a few after eye-opening, but these studies did not report the total amount of synaptic strengthening during glutamatergic waves [41],[42]. So far we explored the segregation outcome with BTDP for initial conditions uniformly distributed in the range 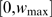 for 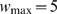. To study the sensitivity to the upper weight bound 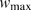, we compared simulations with BTDP using 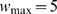 (Figure 6A) and 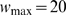 (Figure 6B). We observed no differences in the segregation outcome for initial conditions in the region 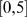 when both ON and OFF segregation occur, in contrast to STDP with subtractive normalization (Figure 4E). The main effect of increasing 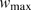 was that simulations took longer to show segregation. Therefore, segregation results with BTDP are robust under changes in the scaling of synaptic weights. Note that the linear model is by design independent of 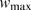, which supports its usefulness in predicting segregation.

### The one-second offset in the firing of ON and OFF RGCs is necessary for segregation

Kerschensteiner and Wong [20] hypothesized that ON and OFF RGCs segregate in the LGN because of the one-second temporal offset in their precisely-ordered firing. We performed simulations with BTDP with the integrate-and-fire model in which we shifted the firing of the ON cells by one second such that it was synchronous with the OFF cells, and found that this abolished segregation (Figure 6C). Analyzing the reduced linear model where the ON/OFF and OFF/ON correlation functions were shifted such that the peak occurred at 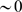 ms, also eliminated segregation (data not shown). This confirms that the temporal asynchrony is a necessary activity cue for the segregation of RGCs of different type, but not a sufficient cue. As we demonstrate next, the correlation structure of the inputs also significantly affects the segregation outcome.

### Insights from the linear model: eigenvalue analysis

We showed that BTDP can explain ON/OFF segregation in the developing LGN of mouse by integrating activity (i) over timescales relevant to the inputs, and (ii) irrespective of the order of pre- and postsynaptic activity. Furthermore, half of the studied data sets (1–3) demonstrated dominance of ON segregation, and the other half (sets 4–6) demonstrated dominance of OFF segregation. To understand why BTDP successfully captured segregation without additional homeostatic mechanisms (but not standard STDP, nor STDP with extended timescales), and to determine which features of the inputs specified ON versus OFF dominance, we dissected the linear model of Equation 7 (Materials and Methods).

**Figure 7:**
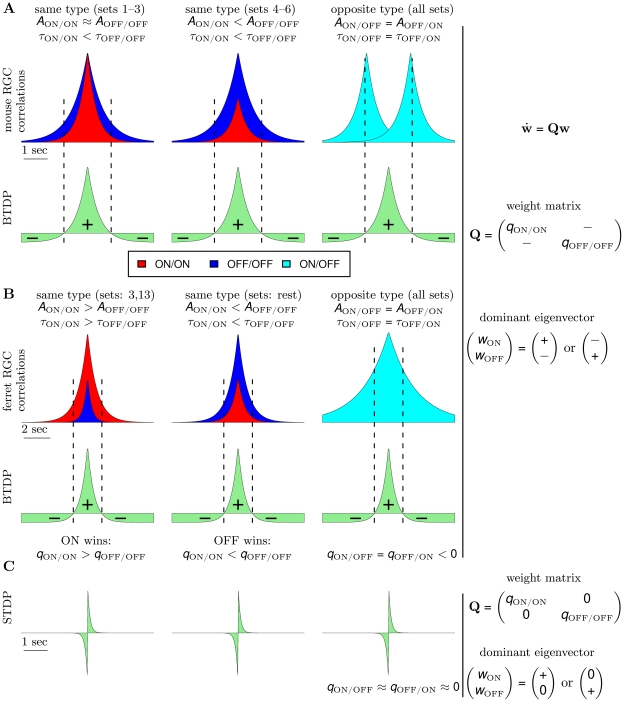
Insights into ON/OFF segregation from the linear model. (A) (left) ON dominance. Factors which determine segregation for data sets 1–3 (data set 1 in Figure 4A) with similar correlation peaks of ON/ON and OFF/OFF pairs. Segregation is the result of a trade-off between the relative areas of the input correlation functions for ON/ON (red) and OFF/OFF (dark blue) pairs integrated by the positive and negative parts of BTDP (green). BTDP favors ON segregation because the smaller area of the ON/ON correlation under the negative part of BTDP (denoted by 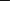) dominates the larger area of the OFF/OFF correlation under the positive part of BTDP (denoted by 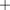). Therefore, 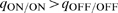 in the plasticity matrix 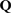 (Equation 1). (middle) OFF dominance. Factors which determine segregation for data sets 4–6 (data set 4 in Figure 4B) with larger correlation peaks of OFF/OFF than ON/ON pairs. BTDP favors OFF segregation because the larger area of the OFF/OFF correlation under the positive part of BTDP dominates the smaller area of the ON-ON correlation under the negative part of BTDP, such that 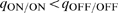 in the plasticity matrix 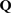 (Equation 1). (right) The reflected (about 0 seconds) input correlations between cells of different type (light blue) have a larger part of their areas under the negative part of BTDP, ensuring negative off-diagonal terms 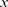 in 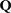 (Equation 1) and introducing competition. (B) (left) Input correlation functions for two of the 15 ferret data sets illustrate dominance of ON segregation. (middle) Input correlation functions for the remaining 13 ferret data sets illustrate dominance of OFF segregation. (right) The input correlation function between cells of different type peaks at 0 seconds for ferret, however, the wide correlation timescale (wider than in mouse) still produces negative off-diagonal terms 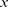 in 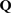 (Equation 1), and correspondingly, competition between the ON and OFF weights. (C) STDP drawn to scale to compare its spike integration time window with the timescale of input correlations shown in (A) and (B). The near-0 off-diagonal terms in 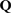 (Equation 3) inhibit segregation.

For comparison, in STDP with subtractive normalization the RGC firing rates dominated segregation due to the millisecond-long integration timescales, while the contribution from the correlations cancelled due to the temporal asymmetry of STDP. In BTDP, however, we show that the RGC correlations dominate segregation due to the matching second-long integration timescales, and are further intensified by the temporal symmetry of BTDP. The entries in the plasticity matrix 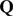 of Equation 8 (Materials and Methods) can be obtained by multiplying the area under the input correlation functions (Figure 1B) and the area under BTDP (Figure 2B). From the decaying exponential fits of the input correlations (Figure 1B and Table 1), we extracted a common feature among the six data sets to be a smaller decay timescale for ON/ON pairs than for OFF/OFF pairs (for data set 6 they are approximately equal), suggesting that ON cells fire shorter high-frequency bursts. On the other hand, after comparing correlation peaks for pairs of different cell types, we found that the six data sets form two groups, which coincide with the preference for ON or OFF dominance (Figure 4C). Data sets 1–3 have similar correlation peaks for ON/ON and OFF/OFF cell pairs (Figure 7A, left), while data sets 4–6 have a higher correlation peak for OFF/OFF pairs than for ON/ON pairs (Figure 7A, middle). Correlation functions for different type RGC pairs are reflections of each other about 0 seconds and have a peak located at 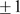 second (Figure 7A, right). Therefore, a significantly larger area of the correlation function of different type RGC pairs falls under the negative part (than under the positive part) of BTDP (Figure 7A, right). This results in negative off-diagonal entries in the plasticity matrix 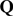 of Equation 8 (Materials and Methods), which are equal due to the reflection about 0 seconds, 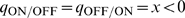:
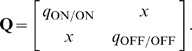
(1)


A matrix of this form has eigenvalues
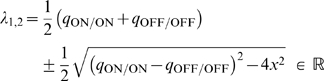
(2)where 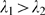 (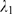 corresponds to the expression with ‘
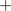
’ and 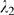 with ‘
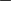
’ sign). The eigenvector, 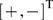, corresponding to the larger eigenvalue 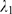 and segregation of the weights, is dominant; the other eigenvector, 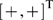, corresponding to 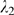 and potentiation of both weights, is suppressed (here, 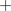 and 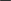 indicate positive and negative entries in each eigenvector, and T denotes transpose). Segregation outcome depends on the relative size of the main diagonal terms in 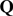. For data sets 1–3 where the peaks for ON/ON and OFF/OFF cell pairs are similar, a larger area of the OFF/OFF correlation (than of the ON/ON correlation) falls under the negative part of BTDP (Figure 7A, left). This gives 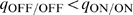 and resulted in dominance of ON segregation in our simulations (Figure 4C). For data sets 4–6, with larger OFF/OFF correlation peaks compared to ON/ON correlation peaks, a larger area of the OFF/OFF correlation (than of the ON/ON correlation) falls under the positive part of BTDP (Figure 7A, middle). This gives 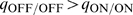 and resulted in dominance of OFF segregation in our simulations (Figure 4C).

Even though this interpretation of the reduced linear model does not directly use the recorded spike trains in each data set (whereas the simulated integrate-and-fire model does), but instead uses fitted estimates of the correlations computed from the most correlated cell pairs, results from the linear and integrate-and-fire models agree (Figure 5). We observed an inconsistency only in data sets 4 and 5 for 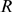 larger than experimentally-observed, where the original correlation peaks and timescales from Table 1 resulted in dominance of ON segregation (data not shown). In this case, the larger area of the OFF/OFF correlation (than of the ON/ON correlation) falling under the positive part of BTDP, is not large enough to counteract the larger area of the OFF/OFF correlation (than of the ON/ON correlation) falling under the negative part of BTDP. To achieve the expected dominance of OFF segregation, we had to increase the decay timescale of the ON/ON correlation (Figure 5B, theory, bottom panel). The simulated integrate-and-fire model using the recorded spike trains (instead of the ON/ON correlations) successfully captured segregation with OFF dominance (Figure 5B, simulation, bottom panel). This suggests that real spike trains may contain higher-order moments which are not always captured by the pairwise correlations of Figure 1B, or that some nonlinear effects of the integrate-and-fire model dominate segregation outcome. Note that this mismatch between the reduced linear and the integrate-and-fire model occurred only for a small range of 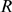 larger than experimentally-observed, and for only two of the six data sets.

In Figure 7C we show a sketch of standard STDP to illustrate the contrast in timescales of spike integration and input correlations. STDP ignores the peaks of the ON/OFF and OFF/ON correlation functions located at 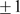 second, and the resulting plasticity matrix has off-diagonal entries close to 0 (for 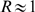)
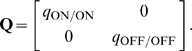
(3)With eigenvectors equal to 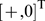 and 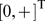, the reduced linear model offers a simple explanation about why STDP failed to capture segregation for 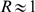 and without normalization constraints. Note that the main diagonal entries are large and positive, due to the contribution from the autocorrelations in Equation 8 for 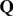 (Materials and Methods), as discussed previously. Increasing 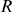 introduced the required asymmetry for competition between the two weights. This follows from the current analysis because large 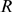 makes the off-diagonal entries in 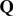 negative as in Equation 1.

Extending integration timescales of STDP to the relevant input correlation timescales results in the plasticity matrix
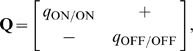
(4)where 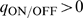 since the ON/OFF correlation peaks at 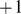 second, while 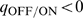 since the OFF/ON correlation peaks at 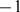 second. This analysis of the modified STDP rule explains why it always resulted in ON segregation in our simulations.

In summary, we have shown that BTDP can explain ON/OFF segregation in the developing mouse LGN driven by spontaneous retinal waves. BTDP promoted cooperation between weights of same type cells and competition between weights of different type cells without additional homeostatic control. We predicted that the LGN neuron will become ON- or OFF-responsive based on a local competition of the firing patterns of neighboring RGCs connecting to it, whose most relevant features for segregation are contained in the pairwise temporal correlations. As ON bursts are shorter than OFF bursts as suggested by the smaller correlation timescales, under BTDP this would suggest dominance of ON segregation. However, if OFF bursts are more highly correlated as suggested by the higher correlation peaks, then the result is dominance of OFF segregation. In particular, we saw that ON and OFF RGCs with sufficiently-different firing properties to compete for the wiring of the LGN neuron, are located close to each other (within 50 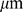). Thus, it is possible that RGC axons which lose the competition at one LGN neuron because of stronger competitors, win at another. This developmental mechanism is similar to that seen in mouse neuromuscular junction where the fate of axonal branches is dependent on the identity of the axons with which they compete [43].

### ON/OFF segregation in ferret

The BTDP model we just described suggests how spontaneous retinal waves can instruct ON/OFF segregation in individual LGN neurons in mouse. Is this result specific to mouse RGCs, or might this model explain segregation in other systems? The firing properties during retinal waves in ferret significantly differ from those in mouse [22]. During the period of ON/OFF segregation in ferret, RGCs of different type fire synchronously, with OFF cells having a significantly higher firing rate than ON cells [21],[22]. In addition, we found that pairs of different type in ferret are correlated over longer timescales than pairs of different type in mouse. In a Hebbian covariance-based model with presynaptic threshold, Lee et al. [22] showed how these patterns can instruct ON/OFF segregation in ferret LGN. We demonstrate that our model with BTDP can also explain ON/OFF segregation in ferret and that it is consistent with the model of Lee et al. [22] (Text S1).

Using the recorded spike trains from the Lee at al. study (15 sets in Dataset S1, each consisting of one ON and one OFF RGC spike train recorded with single electrodes) [22], we simulated the spikes of a postsynaptic LGN neuron using an integrate-and-fire model. As before, synaptic modification was subject to STDP or BTDP, and synaptic weights were bounded between 0 and 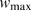. In Table S1, we list the segregation outcome using each plasticity rule. Segregation was more easily achieved than in the mouse model as we only used one ON and one OFF RGC. Thus, as in the reduced linear model, we saw that standard STDP required large ratios of depression-to-potentiation to capture segregation. In contrast, BTDP successfully interpreted firing patterns into segregation for experimental parameters ranges [16].

Earlier we showed that BTDP failed to induce ON/OFF segregation in mice when the one-second firing offset between the ON and OFF cells was eliminated. Although ON and OFF cells fire synchronously in ferret, BTDP could still induce segregation because the correlation between cells of different type in ferret falls off much slower than the correlation between cells of different type in mouse (Figure 7A,B, right and Table 1; also Table S2). We applied a similar analysis to the linear model in Equation 7 (Materials and Methods) as in the previous section. The plasticity matrix 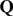 is similar to that for mice with BTDP (Equation 1), with equal and negative off-diagonal entries because the long tails of the ON/OFF correlation function of ferret fall under the negative part of BTDP. As before, the relative sizes of the main diagonal terms in the plasticity matrix 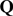, 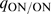 and 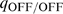, determine the dominance of ON or OFF segregation. Since we only have one ON and one OFF RGCs in each data set, 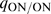 and 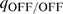 can be computed from the ON and OFF autocorrelations. In two of the 15 data sets (the outliers) we saw a higher correlation peak of the ON autocorrelation (Figure 7B, left), and in the rest of the sets a higher correlation peak of the OFF autocorrelation function (Figure 7B, middle), indicative of the higher firing rate of OFF RGCs (Table S2). In Text S1 we show how our model with BTDP corresponds to the covariance-based model used in Lee et al. [22]. In particular, we make a correspondence between 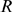 (the depression-to-potentiation ratio in our model with BTDP), and 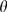 (the presynaptic threshold in [22]) which induced competition between the ON and OFF weights. Due to the higher autocorrelations of OFF RGCs, our model similarly predicted dominance of OFF segregation in 13 of the 15 data sets (and of ON segregation in the other two data sets). To achieve ON segregation in the majority of the sets with OFF dominance, Lee et al. [22] introduced an inhibition term 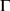. As our model of BTDP using realistic retinal input patterns succeeded in capturing segregation in both mouse and ferret, we suggest that the rules that govern ON/OFF segregation are likely shared between species.

## Discussion

We have used analytical methods and computational simulations to test the hypothesis that spontaneous retinal activity guides the segregation of ON and OFF RGCs in the developing mouse LGN. We have compared two plasticity rules for the development of synaptic weights: STDP and BTDP. Modifications to these rules adapted to the characteristics of the input firing patterns have also been considered. Our results show that STDP alone fails to segregate ON and OFF inputs under realistic ratios of depression-to-potentiation. STDP can segregate mixed inputs when combined with a homeostatic mechanism such as subtractive normalization, however the results are highly sensitive to parameters. By comparison, the recently-proposed BTDP rule [16] robustly segregates ON and OFF inputs in a model LGN neuron by a local comparison of the firing properties of neighboring RGC inputs without requiring normalization. Although ON cells are normally favored to win the competition because they fire before OFF cells, our analytical work indicates that the relative magnitude of the peak correlation of inputs influences which population (ON or OFF) wins the competition. Hence, if OFF RGCs are more strongly correlated than the ON RGCs, they can win the competition. Further, our model with BTDP can also account for segregation of inputs in the developing ferret LGN. Our work also highlights the importance of working with experimentally-recorded spike trains, rather than using synthetically-generated Poisson inputs, as the nature of these correlations can strongly influence developmental outcome.

Twenty years after the initial discovery of retinal waves, there is still an ongoing debate on if, and how, spontaneous activity influences the development of neural connections [7],[8]. The experimental report by Kerschensteiner and Wong [20], on which we have based our modeling, suggests that retinal waves provide an instructive signal during the developmental age addressed. The specific firing patterns of functionally-distinct RGCs they observed are only present during glutamatergic waves and coincide with the period of ON/OFF segregation [44]. Before the switch to glutamatergic waves which occurs as circuits in the developing retina mature, cholinergic waves with different spatio-temporal propagation properties are believed to instruct eye-specific (left-eye/right-eye) segregation [7] (though see [8]). Theoretical models have mostly studied the influence of cholinergic retinal waves upon the development of neuronal connectivity in the context of the eye-specific segregation [16],[45]. As far as we know, our model is the first to address refinements driven by the later-stage glutamatergic waves. While eye-specific segregation in the LGN has been suggested to be driven by summed activity over local regions in the retina [16], our prediction is that more relevant for ON/OFF segregation in the LGN is activity between RGC pairs (as in the reduced linear model). This is likely due to the continued decreased convergence of retinal afferents per LGN neuron during development [6],[41],[42], and receptive field development [39],[46].

In this paper we have taken two complementary approaches to studying segregation of ON and OFF inputs. With simulations, we were able to use the experimentally observed RGC spike trains to generate spiking behavior in a nonlinear integrate-and-fire model; by contrast with a reduced linear model we used only the input correlations of the most correlated cells. The broad similarity in our results between simulation and theory under the more relevant BTDP plasticity rule suggests that the mechanisms for synaptic change guided by spontaneous retinal waves are robust and do not require the precision of individual spikes. Thus, the effect of future manipulations of activity on segregation would not need to be tested with complex spiking models, but predictions can be made with simpler models which use spike-spike correlations over the relevant timescales. Detailed models of retinal wave activity could also help investigate this question, however currently they only model the early cholinergic waves [47]–[49].

Unsurprisingly, we found that a synaptic plasticity rule like STDP, which integrates spikes on much shorter millisecond-long timescales than the relevant timescales of the input correlations, failed to segregate ON and OFF inputs. STDP usually resulted in the strengthening of synaptic weights of both cell types, suggesting a lack of competition. STDP induces competition among synaptic weights when the depression area of the STDP window is slightly larger than the potentiation area (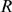 slightly larger than one) under independent Poisson input statistics [25]. Compared to Poisson statistics, real RGC spike trains are highly correlated and can more efficiently drive a postsynaptic neuron. Hence, our model showed that physiologically unrealistic, large depression-to-potentiation ratios were necessary for segregation. When multiple retinal spike trains were used as inputs to an integrate-and-fire model LGN neuron, often even large ratios could not rescue segregation. This was due to the independence in the firing of multiple ON and OFF RGCs over the short timescales of STDP, which resulted in nonselective (same cell type) competition. As an alternative to large depression-to-potentiation ratios, subtractive normalization can induce competition [27],[45]. While this rescued segregation, it was highly sensitive to parameter variation (Figure 4D,E and Table 2). This sensitivity arises as the short integration timescales of STDP fail to reliably sample the noisy (but approximately constant) correlation functions which extend over much longer timescales. Due to the temporal asymmetry of STDP and the mismatch of timescales, potentiation and depression effects cancel, reducing the signaling component of STDP for segregation. Thus, STDP does not utilize the correlations between cells for segregation, but rather the cells' time-averaged firing rates.

In addition to normalization constraints, it is possible that segregation might result from an alternative implementation of a spike-based plasticity rule which involves higher-order spike integration [11]–[13],[50]. While an exhaustive exploration of all forms of STDP is unfeasible, one spiking plasticity rule stands out as promising: a simple spike-coincidence rule which modifies synaptic strength based on the number of coincident pre- and postsynaptic spikes in a 50-millisecond time window, proposed by Butts et al. [16]. Butts et al. [16] showed that in their highly constrained experimental protocol of pairing one-second-long bursts to examine induced plasticity, this rule was equivalent to the proposed BTDP. Implementing such a spike-coincidence rule in our model with the recorded RGC spike trains, however, did not produce segregation results consistent with BTDP (data not shown), suggesting that real spike trains contain higher-order spike dependencies that cannot be captured within a simple spike-coincidence rule.

We believe a burst-based rule is most relevant for retinogeniculate development for several reasons. It reflects the firing patterns of RGCs during spontaneous retinal waves; by contrast, STDP rules have been proposed for mature sensory systems where single spikes can evoke postsynaptic activity [9],[26], or in a developing retinotectal system where activity is visually-evoked [10]. Bursts are more reliable in transmitting information to postsynaptic neurons through immature synapses because of slow and uncertain vesicle release [51]. Additionally, the developmental switch from the NR2B to NR2A subunit composition of NMDA receptors may be responsible for a developmental transition from a second-long burst-based to a millisecond-long spike-based rule governing plasticity, since NR2B has slower kinetics than NR2A [52]. A burst-based rule like BTDP is also consistent with a detailed analysis of retinal wave characteristics, which suggested that correlated high-frequency bursting of neighboring RGCs is the driving factor for retinogeniculate refinements [15]. It is possible, though, that BTDP is a phenomenological interpretation of a spike-based mechanism which can capture second-long input correlations. To investigate such issues further, a better understanding of the biophysical mechanisms which synapses employ to detect bursts, and how they modulate synaptic strength, is needed. To implement BTDP, we detected bursts using burst statistics previously analyzed from the experimental recordings by Kerschensteiner and Wong [20]. Since burst statistics (firing rate, duration, spike frequency) vary during retinal waves, and because synapses cannot extract these statistics off-line, the biophysical mechanisms would need to act as on-line burst detectors. Our model suggests that a simple on-line spike accumulation method (Figure 2C) can detect burst latencies. Although this method will not find the true burst onset, the error is well within the 100 ms limit that has been suggested to preserve information content of retinal waves [14].

In contrast to STDP with subtractive normalization, BTDP utilized the RGC correlations over the relevant second-long timescales to generate segregation without parameter sensitivity (for instance, to 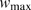). We found that matching the integration timescales of the synaptic plasticity rule with the timescales of input correlations was not the only requirement to achieve robust segregation. When we increased the timescales in STDP, we found that the plasticity rule always favored the cell that fired first, ON. Satisfying both features of long timescales and temporal symmetry of input integration, BTDP successfully accounted for the development of both ON- and OFF-responsive LGN neurons in mouse [39]. Furthermore, while STDP with normalization required careful tuning to match results from the linear and nonlinear postsynaptic models and to produce both ON- and OFF-responsive LGN neurons, BTDP demonstrated consistent segregation irrespective of the modeling approach. With the reduced linear model we could pinpoint how BTDP was able to interpret RGC correlations into robust segregation. The linear model interpretation of BTDP relates to classic models of ocular dominance, where competition was induced by anti-correlated opposite-eye activity [32]. We found that the number of ON and OFF RGCs initially connected to the LGN neuron did not bias the segregation outcome, unlike previously shown [22]. In fact, which cell type won in the LGN depended on a local competition of the firing patterns of neighboring RGCs projecting to the LGN neuron. Additionally, the simulated integrate-and-fire model demonstrated further loss of same-type inputs to the LGN neuron during development: after the segregation of ON and OFF inputs (where all inputs of one type were eliminated), BTDP resulted in additional pruning of the winning inputs to 1–3 as observed in development (Figure 6A, B) [6],[41].

Our model with BTDP also successfully explained ON/OFF segregation in ferret where ON and OFF RGCs fire synchronously, but with a higher firing rate for OFF RGCs than ON [22]. Consistent with a previous covariance-based model (Text S1), segregation was generally in favor of the more active OFF cell. Lee et al. [22] introduced inhibition to allow the less active ON cell to win. Despite the different input firing properties in these two species, our model suggests that ferret and mouse have a similar form of retinogeniculate plasticity and that the synaptic plasticity rules which govern ON/OFF segregation are shared between species.

In addition to segregation of RGCs onto individual LGN neurons, neighboring LGN neurons within a sublamina in ferret respond to the same cell type [38]. By contrast, ON- and OFF-responsive LGN neurons in mouse are distributed in a salt-and-pepper pattern [39]. This difference could be due to molecular cues [53], or more straightforwardly, lateral connectivity between LGN neurons which we could test by modeling a population of postsynaptic neurons. Future modeling would need to address the role of visual activity through closed eyelids together with spontaneous activity from retinal waves in ON/OFF segregation in these species. Dark-rearing in ferrets prevented ON/OFF segregation in the LGN [54]. Our results and those of Lee et al. [22] found that spontaneous activity is sufficient to drive ON/OFF segregation, suggesting that visual activity may accelerate ON/OFF segregation. Thus, it is possible that segregation would still occur in the absence of visually-evoked activity but it would take longer. While the effect of dark-rearing on ON/OFF segregation in the LGN has not been studied in mice, there are conflicting reports on the development of ON/OFF segregation of RGC dendrites [55],[56]. It would be useful to manipulate spontaneous retinal activity without manipulating visual experience, and to examine ON/OFF segregation in dark-reared animals at later ages. This would allow for an independent evaluation of the significance of spontaneous and visually-evoked activity in the retina on ON/OFF segregation.

Experiments which manipulate correlations between RGCs without eliminating spontaneous activity itself have proven extremely useful in answering the key question of whether retinal waves influence development. Recent work reports that mice lacking the 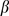
2 subunit of the acetylcholine receptor have significantly altered retinal wave patterns [57],[58], which affect projections in the LGN and the superior colliculus [28],[39],[59]. In addition to stronger correlations between RGCs located further apart in these mutants which are not present in wild type, one study has found a directional bias of wave propagation in wild type animals [58]. The elimination of this bias in 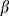
2 mutants is consistent with asymmetric refinement of retinal projections in LGN and superior colliculus [28],[39],[59]. While these findings represent a significant step forward towards understanding the role of retinal activity patterns in development, along with theoretical modeling, they mostly address cholinergic retinal waves [16],[45]. It would be also useful to construct a more complete model for the development of the retinogeniculate pathway in two different stages, at the retinal level capturing the varying spatio-temporal properties of retinal waves, and at the retinogeniculate synapse. If cholinergic and glutamatergic waves are indeed responsible for different aspects of LGN development (eye-specific and ON/OFF segregation, respectively), these studies may provide insights into the mechanisms by which synaptic plasticity rules interpret ongoing changes in the firing patterns of RGCs to explain changes in the synaptic strength of developing connections.

## Materials and Methods

A synaptic weight representing the strength of the connection between the 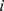-th RGC and the LGN neuron is denoted by the dimensionless quantity 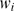, and maintained in the range 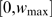. The lower bound ensures that all weights remain excitatory (in line with Dale's Law [60]), and the upper bound prevents the weights from growing arbitrarily large [25],[27]. Table 3 lists the key model parameters used in the two different modeling approaches.

### 

#### Theory: linear model

Instead of using the full set of ON and OFF spike trains from each data set, we used pairwise spike-spike correlations as inputs, and simulated postsynaptic activity with a linear model, following the approach of Kempter et al. [24],[61]. Representing a presynaptic spike train 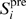 with spikes at times 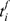 as a sum of delta functions, the activity of a postsynaptic neuron receiving inputs from all such presynaptic trains 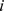, follows a linear Poisson model with an instantaneous time-dependent firing rate (given as an ensemble average), 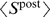


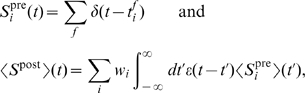
(5)where 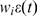 may be interpreted as an excitatory postsynaptic potential (EPSP) representing the probability that the postsynaptic neuron will fire a spike given a presynaptic spike at time 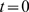, proportional to the weight 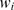. We used
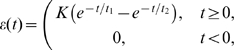
(6)where 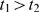, 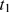 denotes the decay time and 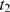 denotes the rise time of the EPSP kernel, and 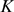 is a normalizing constant such that 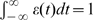. Initially, we tested millisecond timescales for these time constants as recorded in cortical neurons [62]. We also extended these time constants to match recordings for developing LGN neurons [63] (Table 3), but we found no effect on segregation outcome.

Representing the output neuron with a linear Poisson model allows us to write a linear system for the weight dynamics
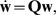
(7)assuming that weight modification is slower than the firing of pre- and postsynaptic spikes, where **Q** is the convolution of the input correlation matrix 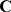 with the plasticity rule 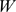, which we call a ‘plasticity’ matrix
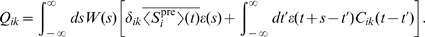
(8)In particular, 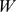 denotes a functional representation of the synaptic plasticity rule we studied, STDP or BTDP (presented below), 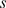 is the timing between a pair of spikes of the inputs 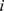 and 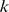, 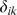 is the Kronecker delta equal to 1 only if 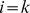, and 0 otherwise, 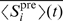 is the mean firing rate of presynaptic RGC 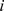 (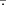 is the temporal average taken over the duration of the recording), and 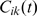 is the input correlation function for the pair of inputs 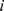 and 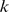


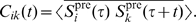
(9)Note that this input correlation involves only presynaptic spike trains, and can be derived by substituting 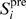 into 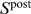 in Equation 5. The input correlation of Equation 9 was computed by binning the number of spikes for the 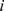-th and 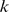-th spike trains into bins of size 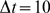 ms and summing the bins over the given time lag 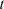.

Instead of studying the evolution of all ON and OFF weights for each data set, we studied a reduced system with a two-dimensional weight vector 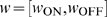 (termed *reduced linear model*), such that segregation occurred whenever one weight potentiated to 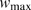 and the other depressed to 0. For the entries of the 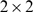 correlation matrix 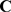 used in 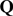 in Equation 8, we took
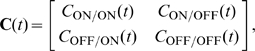
(10)where the correlation functions with the largest peak amplitudes for all pairs of each cell type were selected. We fitted a symmetric decaying exponential to the input correlation function for each RGC pair, 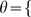
ON/ON, ON/OFF, OFF/ON, OFF/OFF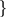:
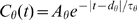
(11)The parameters 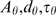 illustrated in Figure 1B were estimated using a nonlinear least squares procedure in R [64], and are reported in Table 1.

In the analysis of STDP, we observed a mismatch between the results from the reduced linear and the nonlinear integrate-and-fire models (Figure 4D, E). To determine the origin of this disagreement, we also implemented a *full linear model* where we used the raw correlations from Equation 9 to obtain the full plasticity matrix 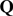 in Equation 8. As we did not observe a difference in the results (Table 2) between the reduced and the full linear model with STDP, and the results of the reduced linear and the nonlinear integrate-and-fire model with BTDP were consistent, we examined only the reduced linear model under BTDP.

#### Simulation: integrate-and-fire model

The retinal wave structure of the inputs manifests itself in LGN neurons as large periodic barrages of postsynaptic currents which drive bursts of action potentials [65]. To replicate realistic LGN firing patterns, we generated postsynaptic activity of the LGN neuron according to a nonlinear quadratic integrate-and-fire model by Izhikevich [66] (though simulating postsynaptic activity with a leaky integrate-and-fire model [25] did not affect our results)
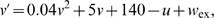
(12)

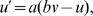
(13)where 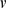 and 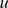 are dimensionless variables, representing respectively, the postsynaptic membrane potential, and a membrane recovery variable providing negative feedback to 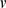. When the membrane potential exceeds 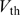, it is reset to 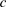, and 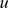 is increased by 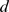; the variable 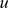 recovers with a timescale of 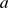, and 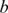 determines the resting potential (parameter values in Table 3). We modeled the synaptic weights 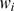 as synaptic conductances, and 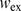 represented the total synaptic conductance received by the postsynaptic LGN neuron [25]. Equations were solved using a forward Euler integration scheme with a fixed time step of 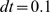 ms, to generate postsynaptic spikes of the same precision as the input spikes.

Unlike the linear model, here we studied the temporal evolution of ON and OFF weights for all inputs in each data set. To compare results with the reduced linear model where a single ON and a single OFF weight were studied, we assigned all ON weights the same initial strength, and all OFF weights the same initial strength (but different from the initial strength for ON). Testing different initial conditions for all 5–8 weights per data set would have required a representation of the weight dynamics in a 5–8-dimensional space. Choosing the same initial condition for all ON and for all OFF weights, we ran a separate simulation for each initial condition combination. Segregation of the inputs was interpreted as the depression of all weights of one cell type, and the maximal potentiation of some weights of the other cell type. As we simulated the dynamics in the full weight space with all available spike trains in each data set, a succinct two-dimensional representation as for the linear model was unfeasible. Note that all ON and OFF inputs in each data were used when that particular data set was explored; spike trains were not mixed between data sets. Total number of retinal afferents in each data set were 5–8 (Table 1), which agrees with data from mouse at P12 [42],[67]. The data sets for both mouse and ferret are provided as supporting material (Dataset S1) with permission from the authors.

#### Plasticity rules: STDP versus BTDP

According to pair-based STDP, synaptic weights were modified based on pairings between pre- and postsynaptic spikes separated by 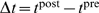, where the order of the spikes determined the sign of synaptic change. We implemented STDP assuming all-to-all interactions such that every presynaptic spike interacted with all previous postsynaptic spikes within the integration timescale, and vice versa [25]. Each spike pair contribution was additive and independent from all others, so the overall synaptic change was the cumulative effect of multiple spike pairings [16],[25] (though see [31] for a review on STDP). STDP (Figure 2A) was formally modeled according to the function
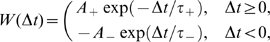
(14)where 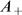 and 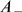 denote the maximum amount of potentiation and depression for 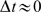, and 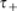 and 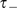 denote the spike integration timescales over which synaptic potentiation and depression are effective. Original STDP experiments report timescales on the order of tens of milliseconds [9],[10],[26], which we tested and also extended (Table 3).

In contrast to STDP, BTDP is temporally-symmetric, such that the timing but not the order of the bursts determines the sign of synaptic change over second-long timescales [16] (Figure 2B). We implemented BTDP following the same approach and assumptions as STDP, except that in 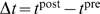, the start times of the pre- and postsynaptic bursts were used for 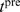 and 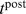.

The function describing synaptic change according to BTDP was experimentally fitted by Butts et al. [16] to a line with a negative (positive) slope for 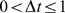 (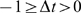) second, and constant depression 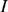 for 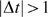 second. To implement BTDP similarly to STDP, we used an exponential representation of BTDP which preserves the timescales and temporal symmetry of burst integration as in the original formulation of BTDP. The exponential approximation is given by
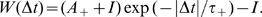
(15)Here, 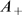 denotes the maximum amount of synaptic potentiation given a pair of precisely synchronous pre- and postsynaptic bursts, 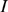 the amount of depression for bursts separated by more than 1 second, and 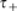 the time constant which controls the burst latency over which potentiation and depression occur.

For the derivation of the linear system with BTDP, we used the spike-spike input correlations as for STDP (statistics given in Table 1), even though BTDP modifies synaptic weights based on a burst latency computed from the start of bursts. This was justified because postsynaptic activity was still generated on the level of spikes (rise in EPSP magnitude at the arrival of a presynaptic spike), and BTDP was robust as long as the timescales of input correlation matched the second-long integration timescales of BTDP (discussed in Results).

To ensure smooth synaptic weight dynamics over time, we used relatively small values of maximum potentiation and depression amplitudes in each rule (Table 3). The resulting slow accumulation of synaptic change required a cyclic presentation of the input spiking patterns (10–50 times) to achieve segregation of the inputs, comparable to the 1–2 day duration of glutamatergic waves corresponding to the period of the highest degree of retinogeniculate refinements. We studied the resulting segregation of inputs as we varied the ratio of depression-to-potentiation, 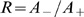 for STDP, and 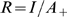 for BTDP (even though the definitions are different, the meaning for 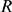 is the same in both rules). Simulation code is available as supporting material ( Protocol S1).

To quantify the degree of segregation, we used the following measure [22]

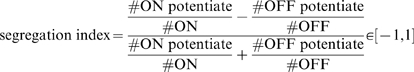
(16)such that index of 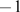 denotes OFF segregation and index of 1 denotes ON segregation. Indices in-between indicate that the LGN neuron responds to mixed ON and OFF inputs.

#### Implementation of subtractive normalization

Subtractive normalization was implemented at the level of individual neurons following findings in superior colliculus, where the number and the strength of retinal afferent synapses received by a postsynaptic neuron was preserved during development [36]. For a total of 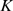 synaptic weights determined by the number of ON and OFF spike trains in each data set with initial conditions in 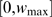, we set the total number of weights maintained by the LGN neuron at maximal strength 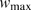 to be 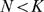. Thus, we made the postsynaptic LGN neuron maintain a target total synaptic strength of 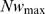 at all times during simulated development. Low retinal convergence into the LGN during adulthood has given estimates for the surviving number of afferents, 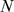, around 1–3 [6],[41],[42]. For each data set we set 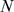 to the smaller number of total ON and OFF weights: we chose 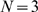 for data set 1 and 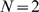 for data sets 2–6; note that for data set 6, 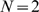 resulted in more consistent segregation results than 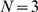. For unbiased initial conditions, the subtractive normalization constraint made all 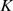 weights start at initial strength of 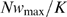. Normalization was applied whenever the plasticity rule modified synaptic strength by 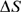 (positive if weights were potentiated, or negative if weights were depressed) changing total synaptic strength such that it deviated from the target 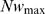. The excess/deficit from this target amount was removed/added to each weight equally. For instance, for a total of 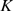 synaptic weights each weight was updated by 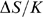. Synaptic weights were still subjected to the hard bounds keeping them within 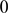 and 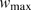.

## Supporting Information

Text S1 A comparison between the model in Lee et al. (2002) and BTDP.
(0.06 MB PDF)

Table S1 Segregation results for ferret using STDP and BTDP.
(0.03 MB PDF)

Table S2 Correlation fits for ferret.
(0.03 MB PDF)

Dataset S1 Spiking data from mouse and ferret.
(0.70 MB ZIP)

Protocol S1 C code for implementing STDP and BTDP.
(0.07 MB ZIP)
