# Supplementary material for: Burst-Time-Dependent Plasticity Robustly Guides ON/OFF Segregation in the Lateral Geniculate Nucleus
Source: PLoS Comput Biol. 2009 Dec 24;5(12):e1000618. doi: 10.1371/journal.pcbi.1000618 (PMC2790088; doi:10.1371/journal.pcbi.1000618)
Supplement: Text S1 — A comparison between the model in Lee et al. (2002) and BTDP. (0.06 MB PDF) [file pcbi.1000618.s001.pdf]

A comparison between the model in Lee et al. [1] and BTDP

Here we discuss in what aspects the model for ON/OFF segregation in ferret presented in [1] is similar to, and how it differs from our model of BTDP. Since the ferret model used the covariance matrix of one ON and one OFF recordings as inputs, we explored consistency with our reduced linear model of BTDP. Denoting the synaptic weight between the i-th RGC and the postsynaptic cell with w_i, Lee et al. [1] represented a small change in w_i as a function of the presynaptic activities x_i and the postsynaptic activity y. The presynaptic threshold parameter θ was used to model an inter-synaptic interaction to induce synaptic competition, giving an equation for the change in w_i

$$\Delta w_i = \eta y(x_i - \theta) \tag{1}$$

(Equation 6 [1]), where η is a small parameter to ensure slow weight modification compared to the rate at which RGCs fire. Ignoring the inhibition term Γ, the equation for postsynaptic activity (Equation 8 [1]) becomes

$$y = \sum_j w_j x_j. \tag{2}$$

Substituting this into Equation 1 above, yields

$$\Delta w_i = \eta \sum_j (x_j x_i - x_j \theta) w_j \tag{3}$$

(Equation 9 without Γ [1]). Averaging over the ensemble of presynaptic activity x_j, this results in (Equation 10 without Γ [1])

$$\Delta w_i \approx \langle \Delta w_i \rangle = \eta \sum_j (\mathbf{C}_{ij} - \overline{x}_j \theta) w_j, \tag{4}$$

where $\mathbf{C}_{ij} = \langle x_j x_i \rangle$ is the raw input correlation matrix and $\overline{x}_j$ is the average activity of x_j.

To show how the form of Equation 4 compares to our linear system for ON/OFF segregation under BTDP, we let

$$\mathbf{Q}^* = \mathbf{C} - \overline{\mathbf{x}} \, (\theta \mathbf{n})^{\mathrm{T}}, \tag{5}$$

where $\overline{\mathbf{x}} = (\overline{x}_1, \overline{x}_2)^{\mathrm{T}}$, $\mathbf{n} = (1,1)^{\mathrm{T}}$ and T denotes the transpose. Then Equation 4 becomes

$$\dot{\mathbf{w}} = \mathbf{Q}^* \mathbf{w}. \tag{6}$$

This corresponds to Equation 7 (Materials and Methods) in the our linear model of BTDP with plasticity matrix $\mathbf{Q}$ given by Equation 8 (Materials and Methods). Expanding the 2×2 covariance matrix $\mathbf{Q}^*$ for the ON and OFF weight dynamics in ferret,

$$\mathbf{Q}^* = \begin{pmatrix} \langle x_1 x_1 \rangle - \overline{x}_1 \theta & \langle x_2 x_1 \rangle - \overline{x}_1 \theta \\ \langle x_1 x_2 \rangle - \overline{x}_2 \theta & \langle x_2 x_2 \rangle - \overline{x}_2 \theta \end{pmatrix} \tag{7}$$

we can make a direct correspondence between the role of R in the plasticity matrix $\mathbf{Q}$ (Equation 1

in Results and Figure 7A,B), and the role of θ in $\mathbf{Q}^*$. As we previously reported (see Results), the competition between the ON and OFF weights in the linear model of BTDP arises due to the negative off-diagonal terms in the plasticity matrix $\mathbf{Q}$ (Equation 1, Results). Since the larger area of the ON/ON and OFF/OFF input correlations (Figure 7A, left and middle) falls under the positive part of BTDP, the main diagonal entries $q_{\mathrm{ON/ON}}$ and $q_{\mathrm{OFF/OFF}}$ of $\mathbf{Q}$ (Equation 1, Results) are always positive and determine which weight wins the competition. In a similar fashion, we can show that the matrix $\mathbf{Q}^*$ in the Lee et al. [1] model has negative off-diagonal entries inducing weight competition, and positive main diagonal entries determining the winning weight. Lee et al. [1], showed that the same-cell type correlation matrix elements are larger than the different-cell type correlation matrix elements in $\mathbf{C}$ (Table 1 [1])

$$\mathbf{C}_{ii} > \mathbf{C}_{ij} \text{ and } \mathbf{C}_{ii} > \mathbf{C}_{ji} \qquad (\text{with } \mathbf{C}_{ij} = \mathbf{C}_{ji} \text{ and } i \neq j).$$

Therefore, subtracting the amount $\overline{x}_i\theta$ from row i in $\mathbf{C}$ gives positive terms on the main diagonal (due to the larger entries C_{ii}), and negative terms otherwise (due to the smaller off-diagonal entries C_{ij}). Thus, the main diagonal entries in $\mathbf{Q}^*$ are positive and the off-diagonal entries are negative, i.e.

$$\mathbf{C}_{ij} - \overline{x}_j\theta > 0 \text{ for } i = j, \text{ and } \mathbf{C}_{ij} - \overline{x}_j\theta < 0 \text{ for } i \neq j.$$

With this formulation, the off-diagonal terms in $\mathbf{Q}^*$ of the Lee et al. [1] model are not equal as the terms x in $\mathbf{Q}$ of our linear model with BTDP (even though $C_{ij} = C_{ji}$, a different amount $\overline{x_j}\theta$ was subtracted from each C_{ij}). In this sense, our linear model of BTDP differs from the covariance-based model of Lee et al. [1]. As we discussed in Results, and in agreement with models of ocular dominance segregation, however, competition between the ON and OFF weights arises provided the off-diagonal entries in the plasticity matrix $\mathbf{Q}$ or $\mathbf{Q}^*$ are negative [2]. Which cell wins the competition depends on the dominant term on the main diagonal in the plasticity matrix. Both of these conditions hold in our model with BTDP and in the Lee et al. [1] model. For the mouse data, we found a natural division among the sets (Table 1): some sets showed dominance of ON segregation (1–3), while others showed dominance of OFF segregation (4–6). For the ferret data, however, the segregation outcome was in favor of the more frequently-firing OFF cell (Table S1), consistent with the model in Lee et al. [1]. To allow for ON segregation (as LGN neurons are both ON- and OFF-responsive [3]), Lee at al. [1] introduced an inhibition term Γ which silenced the more-active (OFF) cell allowing the less-active (ON) cell to win. The functional implications of this term are further discussed in [1].

This text demonstrates how different plasticity rules can be reduced to the same mechanism to explain ON/OFF segregation in two different species. We showed that a simple covariance-based Hebbian plasticity rule with an interaction term θ which can explain ON/OFF segregation in ferret, can be related to a realistic plasticity rule, BTDP, which successfully captures ON/OFF segregation in both mouse and ferret. This suggests that the rules which govern ON/OFF segregation may be shared between species.

References

1. Lee CW, Eglen SJ, Wong ROL (2002) Segregation of ON and OFF retinogeniculate connectivity directed by patterned spontaneous activity. J Neurophysiol 88: 2311–2321.

2. MacKay DJC, Miller KD (1990) Analysis of Linsker's application of Hebbian rules to linear networks. Network 1: 257-297.

3. Grubb MS, Rossi FM, Changeux JP, Thompson ID (2003) Abnormal functional organization in the dorsal lateral geniculate nucleus of mice lacking the $\beta 2$ subunit of the nicotinic acetylcholine receptor. Neuron 40: 1161–1172.
