# Supplementary material for: Burst-Time-Dependent Plasticity Robustly Guides ON/OFF Segregation in the Lateral Geniculate Nucleus
Source: PLoS Comput Biol. 2009 Dec 24;5(12):e1000618. doi: 10.1371/journal.pcbi.1000618 (PMC2790088; doi:10.1371/journal.pcbi.1000618)
Supplement: Table S1 — Segregation results for ferret using STDP and BTDP. (0.03 MB PDF) [file pcbi.1000618.s002.pdf]

Table S1. Segregation results using a numerical simulation of STDP ($\tau_+ = \tau_- = 20$ ms) [1] and BTDP ($\tau_+ = 500$ ms) [2] for 15 data sets of retinal wave activity in ferret (P16-P24) studied by Lee et al. [3]. Postsynaptic activity of the LGN neuron was generated using the quadratic integrate-and-fire neuron by Izhikevich [4]. This table lists the minimum depression-to-potentiation ratio $R = A_-/A_+$ in STDP needed for segregation, a range of the depression-to-potentiation ratios $R = I/A_+$ in BTDP which resulted in segregation, as well as the cell type which won the competition in each case. For set 4, different ranges of R in BTDP resulted in two segregation outcomes.

Set Number	min R for STDP	STDP outcome	Range of R for BTDP	BTDP outcome
1	1.8	OFF	0.01-0.15	OFF
2	2.5	OFF	0.13-0.30	OFF
3	1.7	ON	0.05-0.45	ON
4	2.0	OFF	0.05-0.27	OFF
			0.28-0.45	ON
5	1.8	OFF	0.07-0.26	OFF
6	1.5	OFF	0.03-0.30	OFF
7	6.6	OFF	0.03-0.20	OFF
8	2.1	OFF	0.11-0.25	OFF
9	1.8	OFF	0.03-0.28	OFF
10	1.9	OFF	0.10-0.32	OFF
11	3.5	OFF	0.09-0.35	OFF
12	2.5	ON	0.25-0.35	OFF
13	2.4	ON	0.05-0.29	ON
14	2.1	OFF	0.05-0.25	OFF
15	3.6	OFF	0.01-0.59	OFF

References

1. Song S, Miller KD, Abbott LF (2000) Competitive Hebbian learning through spike-timing-dependent synaptic plasticity. Nat Neurosci 3: 919–926.
2. Butts DA, Kanold PO, Shatz CJ (2007) A burst-based "Hebbian" learning rule at retinogeniculate synapses links retinal waves to activity-dependent refinement. PLoS Biol 5: 0651–0661.
3. Lee CW, Eglen SJ, Wong ROL (2002) Segregation of ON and OFF retinogeniculate connectivity directed by patterned spontaneous activity. J Neurophysiol 88: 2311–2321.
4. Izhikevich E (2003) Simple model of spiking neurons. IEEE Trans Neur Net 14: 1569–1572.
