# Supplementary material for: Burst-Time-Dependent Plasticity Robustly Guides ON/OFF Segregation in the Lateral Geniculate Nucleus
Source: PLoS Comput Biol. 2009 Dec 24;5(12):e1000618. doi: 10.1371/journal.pcbi.1000618 (PMC2790088; doi:10.1371/journal.pcbi.1000618)
Supplement: Table S2 — Correlation fits for ferret. (0.03 MB PDF) [file pcbi.1000618.s003.pdf]

Table S2. Peak amplitudes and decay time constants of the symmetric fall-off exponential fits to the correlation functions for each input pair in each data set from spontaneous retinal activity recordings in ferret [1], fitted using a nonlinear least squares routine in R (values given as estimate ± standard error). Since each data consisted of one ON and one OFF recording, the same type cell correlations are auto-correlations and are all centered at 0. Therefore $d_{\mathrm{ON/ON}} = d_{\mathrm{OFF/OFF}} = 0$ in the table are not listed. Labels are similar to Table 1 (main text).

Set Number	$A_{\mathrm{ON/ON}}$	$\tau_{\mathrm{ON/ON}}$	$A_{\mathrm{OFF/OFF}}$	$\tau_{\mathrm{OFF/OFF}}$	$A_{\mathrm{ON/OFF}}$	$\tau_{\mathrm{ON/OFF}}$	$d_{\mathrm{ON/OFF}}$
1	0.010 ± 0.001	1.126 ± 0.096	0.237 ± 0.004	21.658 ± 3.084	0.021 ± 0.001	7.797 ± 0.651	0.215 ± 0.116
2	0.230 ± 0.005	0.725 ± 0.023	0.163 ± 0.004	1.696 ± 0.051	0.121 ± 0.003	1.775 ± 0.061	-0.005 ± 0.020
3	0.450 ± 0.008	0.506 ± 0.012	0.009 ± 0.001	0.218 ± 0.020	0.035 ± 0.001	0.576 ± 0.021	-0.229 ± 0.011
4	0.594 ± 0.010	0.640 ± 0.015	0.486 ± 0.010	2.590 ± 0.083	0.323 ± 0.003	1.943 ± 0.029	-0.424 ± 0.013
5	0.006 ± 0.000	0.335 ± 0.030	0.018 ± 0.002	1.107 ± 0.073	0.006 ± 0.000	0.807 ± 0.054	0.278 ± 0.027
6	0.009 ± 0.000	0.538 ± 0.030	0.081 ± 0.002	3.505 ± 0.169	0.013 ± 0.000	2.520 ± 0.113	0.057 ± 0.047
7	0.016 ± 0.001	0.513 ± 0.036	0.050 ± 0.001	3.119 ± 0.151	0.010 ± 0.000	3.266 ± 0.200	-0.977 ± 0.072
8	0.190 ± 0.003	3.810 ± 0.102	0.186 ± 0.003	7.015 ± 0.312	0.151 ± 0.002	6.702 ± 0.303	0.529 ± 0.063
9	0.047 ± 0.001	1.080 ± 0.038	1.039 ± 0.020	6.802 ± 0.373	0.156 ± 0.002	1.392 ± 0.025	0.080 ± 0.013
10	0.057 ± 0.001	0.652 ± 0.022	0.201 ± 0.004	1.543 ± 0.042	0.085 ± 0.001	1.249 ± 0.028	-0.208 ± 0.014
11	0.514 ± 0.006	0.839 ± 0.014	0.061 ± 0.003	3.433 ± 0.292	0.106 ± 0.002	2.571 ± 0.070	-1.143 ± 0.028
12	0.077 ± 0.002	1.071 ± 0.035	0.645 ± 0.008	1.284 ± 0.021	0.166 ± 0.003	1.561 ± 0.042	-0.432 ± 0.020
13	0.054 ± 0.002	1.963 ± 0.113	0.007 ± 0.000	0.781 ± 0.061	0.011 ± 0.000	1.660 ± 0.093	-0.152 ± 0.045
14	0.018 ± 0.001	0.502 ± 0.026	0.094 ± 0.002	1.948 ± 0.055	0.022 ± 0.001	1.264 ± 0.054	-0.055 ± 0.027
15	0.005 ± 0.000	0.907 ± 0.090	0.241 ± 0.008	2.624 ± 0.131	0.014 ± 0.000	4.332 ± 0.272	0.325 ± 0.081

References

1. Lee CW, Eglen SJ, Wong ROL (2002) Segregation of ON and OFF retinogeniculate connectivity directed by patterned spontaneous activity. J Neurophysiol 88: 2311–2321.
