# Supplementary material for: Burst-Time-Dependent Plasticity Robustly Guides ON/OFF Segregation in the Lateral Geniculate Nucleus
Source: PLoS Comput Biol. 2009 Dec 24;5(12):e1000618. doi: 10.1371/journal.pcbi.1000618 (PMC2790088; doi:10.1371/journal.pcbi.1000618)
Supplement: Dataset S1 — Spiking data from mouse and ferret. (0.70 MB ZIP) [file pcbi.1000618.s004.zip › DatasetS1/README_data.pdf]

Spiking data from mouse and ferret

Supporting material for Gjorgjieva et al. manuscript: *Burst-time-dependent plasticity robustly guides ON/OFF segregation in the lateral geniculate nucleus* published in PLoS Computational Biology
For further information email Julijana Gjorgjieva at jg447@cam.ac.uk

All times are given in seconds. Each column represents the spiking times of an individual retinal ganglion cell recorded with a multielectrode array. Since not all RGCs within a data set have the same number of spikes, the total number of rows is made equal to the longest spike train, while the shorter ones have been padded with 0's.

For the mouse data (6 sets), each column represents the following cell types:

set 1:	ON	OFF	OFF	ON	OFF	ON		
set 2:	ON	OFF	ON	ON	ON	ON	ON	OFF
set 3:	ON	ON	OFF	OFF	ON			
set 4:	OFF	ON	ON	OFF	ON	ON	ON	
set 5:	OFF	ON	ON	ON	OFF	ON		
set 6:	OFF	OFF	OFF	ON	OFF	ON	OFF	ON

For the ferret data (15 data sets), the first column is always an ON and the second column an OFF cell.

If you re-use these datafiles for your own analysis, please cite the original papers where the data were collected and first published:

- For mouse: Kerschensteiner D, Wong ROL (2008) A precisely timed asynchronous pattern of ON and OFF retinal ganglion cell activity during propagation of retinal waves. Neuron 58: 851–858.
- For ferret: Lee CW, Eglen SJ, Wong ROL (2002) Segregation of ON and OFF retinogeniculate connectivity directed by patterned spontaneous activity. J Neurophysiol 88: 2311–2321.
