# Supplementary material for: Burst-Time-Dependent Plasticity Robustly Guides ON/OFF Segregation in the Lateral Geniculate Nucleus
Source: PLoS Comput Biol. 2009 Dec 24;5(12):e1000618. doi: 10.1371/journal.pcbi.1000618 (PMC2790088; doi:10.1371/journal.pcbi.1000618)
Supplement: Protocol S1 — C code for implementing STDP and BTDP. (0.07 MB ZIP) [file pcbi.1000618.s005.zip › ProtocolS1/README_code.pdf]

# C code for implementing STDP and BTDP

Supporting material for Gjorgjieva et al. manuscript: *Burst-time-dependent plasticity robustly guides ON/OFF segregation in the lateral geniculate nucleus* published in PLoS Computational Biology
For further information email Julijana Gjorgjieva at jg447@cam.ac.uk

To implement STDP, run the file 'STDP.c' separately for each data set. The version available online is set up to run for set 1. To run the code for a different data set, at the top of the file one must change the following:

**total_time** - denotes the total duration (in seconds) of the spiking in the presynaptic neurons. This number will change for the different data sets, such that for the mouse data sets it is 4100 for sets 1 and 4, 2400 for set 2, 2700 for set 3, and 3200 for sets 5 and 6.

**ex** - denotes the number of presynaptic neurons in the data set. For sets 1 and 5 there are six presynaptic neurons, for sets 2 and 6 there are eight presynaptic neurons, for set 3 there are five presynaptic neurons, and for set 4 there are seven presynaptic neurons.

**spike_l** - denotes the total number of recorded spikes per cell in each data set (the spike train length). Since the RGCs in each data sets have a different number of spikes and the shorter spike trains are padded with 0's, this number is the longest spike train in a data set. For set 1 it is 12749 spikes, for set 2 it is 1641 spikes, for set 3 it is 3091 spikes, for set 4 it is 13105 spikes, for set 5 it is 7221 spikes, and for set 6 it is 8837 spikes.

**nfile** - inside the **main** function (line 510) the text has to be changed for the data set currently being run. For instance, for data set 1, the line reads:

```
nfile = fopen("mouse_set1.txt","r");
```

**g[qq]** - initial conditions for the weights are set up in lines 516–523 inside the main function. First in the loop on line 519 the weights are all set to the initial condition for ON, and then on lines 521-523 the weights corresponding to the OFF inputs are set to the initial condition for OFF. For set 1, RGCs in the second, third and fifth columns were OFF, thus g[1], g[2] and g[4] were set to OFF. For the other data sets, they would have to be changed (see the README_data file which specifies which cells are ON and which are OFF in each data set).

Other parameters at the start of the file are currently hard-coded but can be changed if desired:

**dt** - the time step of the simulation in seconds, at the top of the C file. Currently it is set to 0.1 ms, equal to the precision of the presynaptic spikes.

**max_repeats** - the maximum number of times the data set is repeated to ensure synaptic change has reached a steady state (currently set to 50). The simulation will automatically stop when all the weights have reached within 1% of the lower bound (0) and the upper bound ($w_{\max}$), even if the maximum number of repeats has not been reached.

**integrate-and-fire parameters** - parameters $a$, $b$, $c$, $d$ and Vth for the integrate-and-fire model following Izhikevich (2003).

To run the code, the user compiles the file with a C compiler and then needs to input the following parameters (see Table 2 of the manuscript):

**tau_p** - timescale for potentiation in STDP, $\tau_+$.

**tau_m** - timescale for depression in STDP, $\tau_-$.

**A_p** - maximum amount of potentiation in STDP, $A_+$.

**A_m** - maximum amount of depression in STDP, $A_-$.

**w_max** - the upper bound for the weights, $w_{\max}$.

**ON** - initial condition for the ON weights, $w_{\mathrm{ON}}$.

**OFF** - initial condition for the OFF weights, $w_{\mathrm{OFF}}$.

**maintain_syn** - the number of inputs $N$ to be maintained by the postsynaptic neuron in the case of normalization (see main text, Materials and Methods).

**norm_type** - the type of normalization. If 0, then no normalization, if a number $> 0$ then divisive normalization, if a number $< 0$ then subtractive normalization.

The C file produces two .txt documents, one for the temporal evolution of the weights which contains in its name the parameters supplied by the user (up to 6 decimal points)

```
weights_tp_20.000000_tm_20.000000_Ap_0.000100_Am_0.000100_     (...cont'd on next line)
          wmax_5.000000_ON_3.000000_OFF_3.000000_syn_3.txt
```

where each weight is recorded as a separate column every 1 second of simulated time, and another file which contains the spike times of the postsynaptic neuron

```
spikes_tp_20.000000_tm_20.000000_Ap_0.000100_Am_0.000100_      (...cont'd on next line)
         wmax_5.000000_ON_3.000000_OFF_3.000000_syn_3.txt
```

If normalization was implemented, then the file names are preceded with 'subnorm_' for subtractive normalization and 'divnorm_' for divisive normalization.
To implement BTDP, run the file 'BTDP.c' separately for each data set. The version available online is set up to run for set 1. To run it for a different data set, make the same changes as for STDP. Two parameters determine how bursts are detected (both declared at the start of the file), one is min_spikes which is set to 4, denoting the minimum number of spikes in a burst. The other parameters is max_ISI which is set to 0.4 and denotes the maximum allowed interspike interval in a burst. The user inputs are similar as for STDP:

**tau_p** - timescale for potentiation and depression in BTDP, $\tau_+$.

**A_p** - maximum amount of potentiation in BTDP, $A_+$.

**I** - a fixed amount of depression in BTDP, $I$.

**w_max** - the upper bound for the weights, $w_{\max}$.

**ON** - initial condition for the ON weights, $w_{\mathrm{ON}}$.

**OFF** - initial condition for the OFF weights, $w_{\mathrm{OFF}}$.

The C file produces four .txt documents. Two are similar as for STDP, one for the temporal evolution of the weights which contains in its name the parameters supplied by the user (up to 6 decimal points)

```
BTDP_weights_tp_20.000000_tm_20.000000_Ap_0.000100_Am_0.000100_     (...cont'd on next line)
               wmax_5.000000_ON_3.000000_OFF_3.000000_syn_3.txt
```

where each weight is recorded as a separate column every 1 second of simulated time, and another file which contains the spike times of the postsynaptic neuron

```
BTDP_spikes_tp_20.000000_tm_20.000000_Ap_0.000100_Am_0.000100_      (...cont'd on next line)
              wmax_5.000000_ON_3.000000_OFF_3.000000_syn_3.txt
```

The other two files are

**pre_file.txt** - records the index of the presynaptic cell, the start time of a burst, the end time of a burst and the number of spikes in that burst, in a separate row for each burst as time increases.

**post_file.txt** - records the start time of a burst, the end time of a burst and the number of spikes in that burst for the postsynaptic cell, in a separate row for each burst as time increases.
